# The Hallmarks of Flavonoids in Cancer

**DOI:** 10.3390/molecules26072029

**Published:** 2021-04-02

**Authors:** Luis Gustavo Saboia Ponte, Isadora Carolina Betim Pavan, Mariana Camargo Silva Mancini, Luiz Guilherme Salvino da Silva, Ana Paula Morelli, Matheus Brandemarte Severino, Rosangela Maria Neves Bezerra, Fernando Moreira Simabuco

**Affiliations:** 1 Multidisciplinary Laboratory of Food and Health (LabMAS), School of Applied Sciences (FCA), University of Campinas (UNICAMP), Limeira, São Paulo 13484-350, Brazil; lg_saboia@hotmail.com (L.G.S.P.); isadora.bpavan@gmail.com (I.C.B.P.); marianamancini5@gmail.com (M.C.S.M.); luizsalvino@yahoo.com.br (L.G.S.d.S.); apm.morelli@gmail.com (A.P.M.); matheusbr.severino@gmail.com (M.B.S.); rosangelabezerra02@hotmail.com (R.M.N.B.); 2 Laboratory of Signal Mechanisms (LMS), School of Pharmaceutical Sciences (FCF), University of Campinas (UNICAMP), Campinas, São Paulo 13083-871, Brazil

**Keywords:** flavonoids, cancer, cell signaling

## Abstract

Flavonoids represent an important group of bioactive compounds derived from plant-based foods and beverages with known biological activity in cells. From the modulation of inflammation to the inhibition of cell proliferation, flavonoids have been described as important therapeutic adjuvants against several diseases, including diabetes, arteriosclerosis, neurological disorders, and cancer. Cancer is a complex and multifactor disease that has been studied for years however, its prevention is still one of the best known and efficient factors impacting the epidemiology of the disease. In the molecular and cellular context, some of the mechanisms underlying the oncogenesis and the progression of the disease are understood, known as the hallmarks of cancer. In this text, we review important molecular signaling pathways, including inflammation, immunity, redox metabolism, cell growth, autophagy, apoptosis, and cell cycle, and analyze the known mechanisms of action of flavonoids in cancer. The current literature provides enough evidence supporting that flavonoids may be important adjuvants in cancer therapy, highlighting the importance of healthy and balanced diets to prevent the onset and progression of the disease.

## 1. Introduction

Flavonoids represent the largest group of polyphenols found in plant-based foods, including fruits, vegetables, grains, and herbs, as well as in beverages such as tea, wine, and juices [1]. In plants, flavonoids play the role of secondary metabolites, acting as protectors against biotic and abiotic threats, particularly in the defense against ultraviolet radiation and pathogen action. Additionally, they also actively participate in odor, flavor, and color determination in several species [2,3]. The concentration of flavonoids in food is related to several factors, including the variety of the phylum, order, family, and/or species of the plant, as well as the characteristics related to plantings, such as the type of soil, the climatic conditions of the region and the level of maturation of the food. Flavonoids′ concentration and composition also vary depending on the different parts of the plant. Leaves and the peels of fruits are commonly rich sources of flavonoids due to increased susceptibility to stress [4,5,6].

These compounds are composed of fifteen carbon atoms in their chemical structure (Table 1), presenting two benzene rings (A and B) connected through a heterocyclic ring containing oxygen (C). Flavonoids can be subdivided into flavones, isoflavones, flavanones, flavonols, anthocyanidins, and flavans. These classes differ from one another according to the oxidation state of the central carbon in the chemical backbone. Table 1 presents the chemical structures and the main food sources of these subclasses of flavonoids [7].

According to Hanahan and Weinberg [14], cancer may be defined as a heterogeneous disease presenting characteristics related to the uncontrolled growth, division, and invasion of cells in other tissues and organs. Cancer cells undergo important biological changes that allow the emergence of new cellular characteristics, which are known as the hallmarks of cancer: maintenance of proliferative signaling; inactivation of growth suppressors; apoptosis resistance; increase in replication potential; angiogenesis induction; invasion of surrounding tissues and metastasis.

In human nutrition, flavonoids are associated with reduced oxidative stress through the balance between oxidizing and antioxidant molecules and the protection against changes in cell lipids, proteins, and DNA. The administration of diets based on foods rich in phenolic compounds suggests that flavonoids can protect against the development of several types of diseases, including cancer [15,16,17]. However, further studies in humans are needed since most of them are carried out in vitro or in animals. The development of these studies takes place through clinical trials that are classified as the gold standard in the evaluation of therapeutic and preventive actions in health [18]. Currently, several clinical studies are being developed considering flavonoids in cancer therapy (Table 2). Those who reached the final stage showed promising results in the use of these compounds as an auxiliary therapy in the treatment or prevention of various types of cancer. Therefore, more studies are necessary to obtain answers that have not yet been clarified regarding this subject.

Bioavailability is a complex process and depends on several factors, including stability of the digestive system (adequate concentration of enzymes and pH), the composition of the food matrix, and the initial concentration of the compound [27,28]. Most flavonoids are ingested in the usual diet in the form of glycosides, which have one or more sugar molecules linked to phenolic groups or the C-3 hydroxyl group [28]. Normally, these compounds can be absorbed in two ways: the first and simplest is related to the forms of aglycones, which are absorbed directly in the small intestine; the second involves substances found in their glycosylated form, which undergo metabolic reactions of biotransformation (phase I), including oxidation, reduction, and hydrolysis reactions. These compounds are affected by the action of Lactase-Phlorizin Hydrolase (LPH) or Cytosolic β-Glucosidase (CBG), present in intestinal epithelial cells, causing the separation and release of the phenolic compound in an aglycone form. After absorption, the compounds are metabolized by enzymes in the intestinal or liver cells that promote conjugation reactions with methyl, sulfate, and glucuronic acid groups (phase II). These conjugated metabolites are then transported to the bloodstream or returned to the digestive system through enterohepatic recirculation. The absorption of the phenolic compound may be low in the upper gastrointestinal tract. The compounds that were not absorbed in the small intestine pass into the large intestine, where they are metabolized by bacteria present in the microbiota and then can be absorbed or excreted in the feces [29,30,31]. Once absorbed, the flavonoids may have their systemic or local effects on different cell types and biological processes.

It is worth mentioning that there are limitations regarding the use of flavonoids, mainly due to their low bioavailability and toxicity in the human body. Regarding the bioavailability process, the concentration of flavonoids in food does not necessarily correspond directly to the actual amount absorbed and metabolized in the body, which can lead to a reduction of the beneficial effect on health [32,33]. As for toxicity, when flavonoids are ingested in the form of supplements in exorbitant amountss, they can induce pro-oxidant activity, mitochondrial toxicity (potential apoptosis-inducing properties), and interactions with metabolic enzymes [34,35]. Currently, the development of nanoformulations is an alternative to reduce these limitations [36], however, studies targeting methods to improve bioavailability, as well as the concentrations that avoid flavonoid toxicity, should be further investigated.

**Figure 1:**
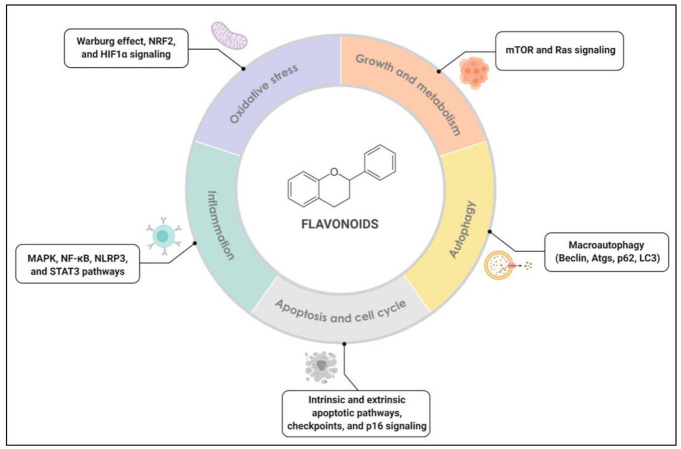
The molecular actions of flavonoids in cell signaling in cancer. Flavonoids significantly alter the biological processes involved in cancer, including inflammation and immunity, redox metabolism, cell growth, autophagy, apoptosis, and cell cycle. Signaling pathways related to these biological processes, which include Mitogen-Activated Protein Kinases (MAPK), Nuclear Factor-κB (NF-ĸB), Nod-Like Receptor Pyrin domain containing 3 (NLRP3) inflammasome, Signal Transducer and Activator of Transcription 3 (STAT3) pathway, Warburg effect, Nuclear Factor Erythroid 2-related Factor 2 (NRF2), Hypoxia-Inducible Factor 1 alpha (HIF1α), mechanistic Target Of Rapamycin (mTOR), Ras, macroautophagy, cell cycle checkpoints, intrinsic and extrinsic apoptotic pathways, and senescence, are modulated by flavonoids in cancer and highlighted in this figure. Original figure made for this review using the Biorender software.

In this context, this review aims to clarify how flavonoids act in the modulation of several biological signaling pathways and defensive systems involved in cancer, including inflammation and immunity, redox metabolism, cell growth, autophagy, apoptosis, and cell cycle (Figure 1). Considering the great number of studies in this area, here we present detailed figures demonstrating the participation of different classes of flavonoids in several steps of those signaling pathways. We also highlight the state-of-art research of these compounds, presenting in-depth tables containing molecular mechanisms, compound concentrations, incubation conditions, and experimental models regarding recent in vitro and in vivo studies. We believe that the compilation of this information brought by this review may greatly contribute to future studies in the area.

## 2. Flavonoids, Inflammation and Immunity

Inflammation has been classified as one of the hallmarks of cancer and is involved in the progression of most types of cancer [14]. Innate and adaptive inflammatory responses are mainly mediated by immune system cells, such as macrophages [37]. Thus, in this section, the immunomodulatory and anti-inflammatory properties of flavonoids will be explored by focusing on specific signaling pathways such as Nuclear Factor-κB (NF-κB), Mitogen-Activated Protein Kinases (MAPK), Nod-Like Receptor Pyrin domain containing 3 (NLRP3) inflammasome, and Janus Kinase/Signal Transducer and Activator of Transcription (JAK/STAT). A summary of the actions of flavonoids in inflammation and immunity is presented in Table 3.

### 2.1. The NF-κB Pathway

NF-κB is a transcription factor composed of a family of proteins: RelA (p65), NF-κB1 (p50), RelB, c-Rel, and NF-κB2 (p52), which are combined in different homo and heterodimers [38]. NF-κB activation is mediated by Lipopolysaccharide (LPS) and pro-inflammatory cytokines such as Tumor Necrosis Factor (TNF-α) and Interleukine-1 (IL-1). NF-κB activation generally occurs in tumor microenvironments of most solid cancers and hematopoietic malignancies, which makes this signaling pathway a potential target for cancer therapy [39].

**Figure 2:**
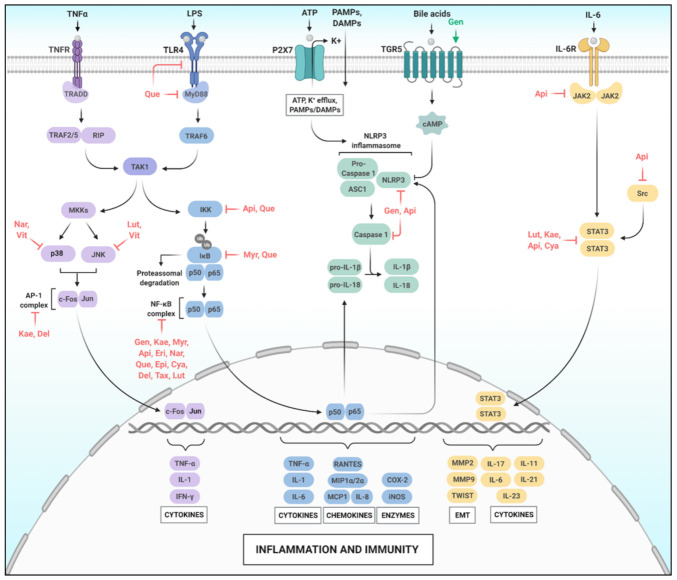
Flavonoids modulate inflammation and immunity pathways in cancer. Pro-inflammatory stimuli like TNF-α and Lipopolysaccharide (LPS) can bind to Tumor Necrosis Factor Receptor (TNFR) and TLR4 receptors, respectively, and activate the TAK1, which is mediated by TNFR-associated Death Domain (TRADD), TRAF2/5, and Receptor-Interacting Protein 1 (RIP) in response to TNF-α/TNFR signaling and Myd88 and TRAF6 in LPS-TLR4 signaling. TAK1 activates Mitogen-Activated Protein Kinases (MKKs) and I Kappa B Kinases (IKKs), leading to translocation of AP-1 and NF-κB to the nucleus. Flavonoids act mainly in inhibiting the nuclear translocation, expression, or phosphorylation of these transcription factors. The first signal for NLRP3 inflammasome activation is through NF-κB-mediated transcription of NLRP3. The second signal is mediated by Adenosine Triphosphate (ATP), Pathogen-Associated Molecular Patterns (PAMPs), Damage-Associated Molecular Patterns (DAMPs), and K+ efflux. When NLRP3 inflammasome is activated, caspase 1 cleaves pro IL-18 and pro-IL-1β in activated forms. Flavonoids act in this pathway mainly by inhibiting caspase 1 activity and reducing the NLRP3 expression. The TGR5-cAMP signaling pathway is activated by Genistein to lead to ubiquitination and degradation of NLRP3. Flavonoids also modulate the JAK2-STAT3 signaling, reducing inflammatory and EMT markers. Epi: Epigallocatechin, Kae: Kaempferol; Myr: Myricetin; Que: Quercetin; Api: Apigenin; Vit: Vitexin; Gen: Genistein; Dai: Daidzein; Lut: Luteolin; Nar: Naringenin; Tax: Taxifolin; Eri: Eriodictyol, Cya: Cyanidin. Original figure made for this review using the Biorender software.

As shown in Figure 2, after Toll-Like Receptor 4 (TLR4) activation by LPS, Myeloid differentiation primary response 88 (Myd88) is recruited to the TLR4 receptor, an important event that activates TNF Receptor-Associated Factor 6 (TRAF6), which in turn activates Transforming growth factor β-Activated Kinase 1 (TAK1). TAK1 activation leads to phosphorylation of I Kappa B Kinase (IKK), an upstream regulator of NF-κB [40]. Tumor Necrosis Factor Receptor (TNFR) activation by TNF-α also recruits Receptor-Interacting Protein 1 (RIP1) and TNF Receptor-Associated Factor 2/5 (TRAF2/5), which are important for TAK1 activation, and consequently activation of NF-κB signaling [41]. I kappa B-alpha (IκB-α) can be phosphorylated, mainly by IKK, causing its ubiquitination and release of NF-κB. The p50/p65 dimer then migrates to the nucleus and regulates gene expression related to immune response, inflammation, cell growth, survival, and development (Figure 2) [42,43]. Classical targets for NF-κB are already described as pro-inflammatory cytokines (TNF-α, IL-1, and IL-6) [44], chemokines (IL-8, Macrophage Inflammatory Protein 1/2 alpha; MIP1α/2α, Monocyte Chemoattractant Protein 1; MCP1, and Regulated upon Activation, Normal T cell Expressed, and Secreted; RANTES) [44,45], Matrix Metalloproteinase 9 (MMP9) [46], proliferation-inducing proteins (cyclin D1 and Myc) [47,48], anti-apoptotic proteins (B-cell lymphoma-extra-large; Bcl-XL, B-cell lymphoma 2; Bcl-2 [49,50], pro-inflammatory enzymes (Cyclooxygenase-2; COX-2 and inducible Nitric Oxide Synthase; iNOS) [43], and angiogenic factors (Vascular Endothelial Growth Factor; VEGF) [51]. Thus, NF-κB is essential for inflammatory responses, linking chronic inflammation and cancer [39,43].

Flavonoids can modulate the NF-κB pathway in different ways [52,53,54,55,56,57,58,59,60,61,62,63,64,65,66,67]. For example, Genistein 27 (Gen-27), the synthetic derivative of Genistein, presents NF-κB inhibitory activity in colon cancer cells when treated with LPS [52]. This compound reduces IκB-α and IKK-α/β phosphorylation and inhibits the nuclear translocation of p65 NF-κB. Gen-27 also decreases the LPS-induced cytokines IL-6 and IL-1β in THP-1 cells [52]. Studies also showed that Kaempferol has anti-inflammatory activities [53,60]. Kaempferol inhibits the DNA binding activity of NF-κB, in addition to decreasing its expression [53]. Another study showed that kaempferol reduces IκB-α/IKK phosphorylation, nuclear translocation of NF-κB, and NF-κB targets gene expression as iNOS, COX-2, TNF-α, IL-1β, and IL-6 in LPS-treated RAW 264.7 macrophages [60].

Myricetin is known to reduce inflammation markers (TNF-α, IL-1β, IL-6, NF-κB, p-NF-κB, and COX-2), thereby preventing chronic inflammation and inflammation-driven tumorigenesis in colon tissue of mice [61]. Eridyctiol, a less studied flavonoid, has also been shown to impact the NF-κB pathway, decreasing the phosphorylation of IκB-α and p65 in U87MG and CHG-5 glioma cells [62]. Other evidence showed that Luteolin decreased the NF-κB activation and also the gene expression of NF-κB targets, such as COX-2, IL-1β, and IL-6 in Phorbol Myristate Acetate (PMA) plus A23187-induced inflammation HMC-1 cells [63].

Studies have shown that Apigenin efficiently regulates the NF-κB pathway [54,64]. Apigenin reduces NF-κB activity in HEK293 cells with TNF-α and IL-1β-induced inflammation, while it decreases IL-6, IL-1β, and TNF-α in macrophages induced by LPS [64]. Shuckla et al. (2015) showed that Apigenin downregulates several NF-κB targets related to proliferation (cyclin D1, and COX-2), angiogenesis (VEGF), and apoptosis (Bcl-2 and Bcl-XL) in Transgenic Adenocarcinoma Mouse Prostate (TRAMP) model [54]. This event is associated with the decrease in IκB-α phosphorylation mediated by inhibiting IKK activation, enabling the suppression of NF-κB activity.

Using an in vivo model of benzo (a) pyrene (B[a]P)-induced lung cancer tumorigenesis, Bodduluru et al. (2016) showed that Naringenin reduces cytokines such as TNF-α, IL-6, and IL-1β while also reducing protein levels of NF-κB, showing a chemopreventive role of Naringenin against chemically induced lung cancer in mice [65]. Taxifolin, a Quercetin derivate, also presents a chemopreventive capacity through modulation of inflammatory pathways [66]. Manigandan et al. (2015) showed that Taxifolin inhibits NF-κB and downregulates COX-2, TNF-α, and cyclin D1, which are known NF-κB targets, in 1,2-Dimethylhydrazine (DMH)-induced mouse colon carcinogenesis [66].

Some studies showed that Quercetin ameliorates the inflammatory process in cancer cells [55,67]. Quercetin presented chemopreventive effects against metastatic cell lines of the human salivary gland, since it increases the expression of IκB-α, an inhibitor of NF-κB activation, and reduces translocation of NF-κB to the nucleus [67]. In HeLa cells, Quercetin reduced IκB-α and IKK-β phosphorylation, NF-κB members (p50 and p65), and cyclin-D1 expression [55].

A study showed that Quercetin reduced the production of nickel-induced cytokines, such as IL-1β, IL-6, TNF-α, and IL-10, in lung cancer cell lines. In addition, Quercetin suppressed the expression of TLR4 and Myd88, in addition to reducing the phosphorylation of IKK-β and IκB-α, the nuclear expression of p65 (NF-κB), and the expression of MMP9 in A549 cells exposed to nickel. Thus, Quercetin presents potential preventive effects in lung cancer [56].

CpG Oligodeoxynucleotides (CpG-ODN), a known TLR9 agonist, can induce the production of several cytokines and metalloproteinases in prostate cancer cell lines [57]. Mukherjee et al. (2014) showed that Epigallocatechin-3-Gallate (EGCG) reverts the inflammation response produced by CpG-ODN, inhibiting cytokines (IL-6, IL-8), chemokines (CXCL1, CCL5, IP-10), metalloproteinase activity (MMP9 and MMP2), and translocation of p65 to the nucleus. Thus, EGCG seems to have a robust anti-inflammatory response, which could be beneficial in prostate cancer treatment [57].

Pratheeshkumar et al. (2014) topically administrated Cyanidin-3-Glucoside (C3G) in SKH-1 hairless mice followed by Ultraviolet (UV) radiation exposure, which usually induces inflammation and photocarcinogenesis in mammalian skin [58]. The results showed that a C3G blocks the NF-κB translocation to the nucleus and reduces UVB-induced COX-2, and iNOS levels. Therefore, C3G may be used against UVB-induced inflammation related to skin disease and skin cancer [58]. Finally, Delphinidin showed anti-proliferative and anti-invasive properties in PMA-induced human breast carcinoma cells (MCF-7) through NF-κB activity inhibition and consequently reduction in MMP-9 expression [59].

### 2.2. The MAPK Pathway

MAPKs are protein-serine/threonine kinases, which include c-Jun N-terminal kinases (JNKs), p38s, and Extracellular Signal-Regulated Kinases (ERKs) [68]. MAPKs are modulated by various signals such as hormones, cytokines, growth factors, and endogenous stress. For this reason, they are also called mitogen and stress-activated MAPKs [69]. As illustrated in Figure 2, similarly to NF-κB activation, MAPK activation is also mediated by TAK1 activation in response to LPS and TNF-α stimulus [70]. TAK1 can activate Mitogen-Activated Protein Kinases (MKKs), which in turn phosphorylates and activates JNK1 and JNK2 [40,70]. Upon activation, JNKs phosphorylate several targets, including the transcription factor c-Jun, which homodimerizes and/or heterodimerizes with c-Fos generating the Activator Protein-1 (AP-1) transcription complex [71,72]. AP-1 can directly bind to AP-1 binding motifs in promoters in the DNA and increase the expression of pro-inflammatory genes, such as TNF-α [73], IL-1 [74], Interferon-gamma (IFN-γ) (Figure 2) [75], and MMPs [76]. The activation of the JNK/AP-1 axis has been involved in the pathogenesis and progression of several cancers [77,78]. A link between this pathway and chronic inflammation-associated cancer development has been proposed [68,69].

p38 is a serine/threonine kinase activated by direct phosphorylation of MKK3 and MKK6 and contributes to AP-1 activation through phosphorylation and activation of Activating Transcription Factor 2 (ATF2) and Ternary Complex Factors (TCFs) [79,80]. p38 has a key role in inflammatory diseases, and its role in cancer is contradictory [81]. p38 may be considered a tumor suppressor, however, some studies also provide evidence for an oncogenic potential related to its pro-inflammatory properties, capacity to regulate epithelial-mesenchymal transition, and angiogenesis [82,83,84,85].

A study showed that Kaempferol reverses LPS-induced inflammation through inhibition of c-fos and decreases the activity of the AP-1 complex in RAW 264.7 macrophages [60]. Luteolin is another flavonoid that possesses an impact on the MAPK pathway. Kang et al. (2010) showed that Luteolin can reduce phosphorylation of JNKs (JNK1, and JNK2) and inflammatory cytokines (TNF-α, IL-8, IL-6) induced by PMA and A23198 in HMC-1 cells [63]. Naringenin inhibits p38, MMP2, and MMP9 activity, blocking migration in glioblastoma cells [86]. Delphinidin is also able to block PMA-induced JNK and p38 MAPK pathways in MCF-7 cells, reducing PMA-induced breast cancer cell invasion. Vitexin, a derivative of Apigenin, also has anti-inflammatory activity through modulation of the MAPK pathway [87]. Rosa et al. (2016) showed that Vitexin reduces neutrophil migration and decreases pro-inflammatory mediators, such as TNF-α, IL-1β, and Nitric Oxide (NO) releases in the peritoneal cavity of LPS-treated mice. In addition, vitexin can reduce TNF-α, IL-1β, NO, Prostaglandin E2 (PGE2) levels and increase IL-10 release in LPS-treated RAW 264.7 cells. Mechanistically, Vitexin inhibits the phosphorylation of p38 and JNK, which explains its described anti-inflammatory effects [87].

### 2.3. The Inflammasome Pathway

Inflammasomes are multimeric protein complexes that are part of the innate immune system. The most studied inflammasome complex is the NLRP3, which consists of the NLRP3 sensor, the Apoptosis-associated Speck-like protein containing CARD (ASC) adapter, and the pro-caspase 1. The NLRP3 sensor has a Pyrin Domain (PYD) in its N-terminal, a central domain of nucleotide-binding and oligomerization (NATCH), and a Leucine-Rich Repeats (LRR) in its C-terminal [88]. The NLRP3 complex is activated by at least two signals. The first involves NF-κB signaling activation, which upregulates pro-IL-1β, pro-IL-18, and NLRP3 protein levels. The second involves extracellular ATP mediated-P2X7 receptor activation, K^+^ efflux, and a set of inflammation-inducing stimuli, such as Pathogen-Associated Molecular Patterns (PAMPs) and Damage-Associated Molecular Patterns (DAMPs) [89,90]. The activation of the NRLP3 inflammasome complex occurs by the oligomerization of NLRP3 mediated by the homotypic interaction between NATCH domains. NLRP3 also interacts with the ASC, an adapter protein, through PYD domains. ASC then recruits cysteine protease pro-caspase-1 through CARD-domain interactions, resulting in autocatalysis and activation of caspase 1 [88]. Pro-IL-1β and pro-IL-18, potent pro-inflammatory cytokines, can be cleaved into biologically active forms of IL-1β and IL-18 by caspase 1, promoting pyroptosis, a form of cell death programmed by inflammation (Figure 2) [91].

The NLRP3 inflammasome is involved with several inflammatory-based diseases, including cancer [92]. Studies have shown that NLRP3 increased expression and/or activity in several types of cancer, such as melanoma [93], head and neck squamous cell carcinoma [94], lung squamous cell carcinoma [95], pancreatic [96], and bladder cancer [97]. Thus, NLRP3 has been investigated as a potential and attractive therapeutic target. Figure 2 illustrates the activation of the NLRP3 inflammasome and summarizes the point actions of flavonoids in this pathway.

Ulcerative colitis (UC), a chronic and inflammatory bowel disease, is an essential factor of colorectal cancer [98]. NLRP3 mediates Dextran Sodium Sulfate (DSS)-induced ulcerative colitis in mice [99]. A study showed that Genistein can inhibit NLRP3 activation and protect DSS-treated mice from ulcerative colitis [100]. In vivo, Genistein also suppressed the production of IL-1β, caspase-1, and the protein level of NLRP3. In macrophages, Genistein inhibited the NLRP3 inflammasome by activating the G-protein coupled bile acid receptor 1 (TGR5)-cAMP signaling pathway, which signals for ubiquitination and degradation of NLRP3 [100]. Thus, Genistein may be an important candidate in the prevention or treatment of ulcerative colitis and colorectal cancer.

Another study showed that Gen-27 is a potent inhibitor of LPS-induced inflammation in RAW264.7 cells, through inhibition of NLRP3 and NF-κB signaling pathways [101]. Gen-27 inhibited nitrite and nitric oxide levels, suppressed the release of pro-inflammatory cytokines, such as TNF-α, IL-1β, IL-6, and IL-18, and decreased the expression of COX-2 and iNOS. In addition, Gen-27 decreased caspase 1 activity, NLRP3 protein levels, and NF-κB (p65) transcriptional activity [101]. Genistein is a promising therapeutic targeting the prevention and therapy of diseases associated with inflammation. Apigenin also reduced LPS-induced inflammation by several mechanisms in human THP-1-derived macrophages [64]. One of them involves inhibition of NLRP3 inflammasome, causing inhibition of caspase 1 activity and IL-1β production [64].

### 2.4. The STAT Family Pathway

Members of the STAT family are signal transducers and activators of transcription [102]. Cytokines such as interleukins, interferons, and peptides hormones can bind to cell surface receptors leading to their homo or heterodimerization. This event causes activation of JAK proteins, which phosphorylate tyrosine residues in the tail of the receptor, thereby creating docking sites for the STAT-receptor interaction. Then, JAK phosphorylates STAT, which is released from the receptor and interacts with other STAT proteins through SH2-domains (Figure 2). JAK-STAT signaling is one of the main pathways in the conversion of the cytokine signal to the response of gene expression, coordinating the proliferation and differentiation of immune cells. STAT1, STAT2, STAT3, STAT4, and STAT6 proteins have pro- or anti-inflammatory properties and are activated by cytokines in inflammation processes [103]. STAT3 is the most studied member of the STAT family. Various cytokines such as IL-11 [104], IL-23 [105], IL-21 [106], IL-6 [107], IL-17 [108], genes related to Epithelial-Mesenchymal Transition (EMT), such as TWIST, MMP2, and MMP9 [109], and angiogenesis such as VEGF [109] are upregulated by STAT3 and reinforce the oncogenic role of this protein.

Some studies have shown that flavonoids can regulate STAT signaling as presented in Figure 2 [60,110,111,112,113,114,115,116,117,118]. Huang et al. (2015) showed that Luteolin inhibits the EMT and metalloproteinase secretion in pancreatic cancer cells (PANC-1 and SW1990) by inhibiting the transactivation of p-STAT3 and transcription mediated by STAT3 [110]. The EMT is associated with the onset of metastasis during tumor progression [119]. Another study showed that Kaempferol alone or in combination with 5-Fluorouracil (5-FU), a chemotherapeutic agent commonly used in colon cancer, reduces STAT3 phosphorylation in both parental and 5-FU chemo-resistant colon cancer cells, concomitant with a decrease in IL-8 and VEGF levels in 5-FU resistant cells [111].

In leukemia cells, HL60, and TF1 cells, Apigenin exerts a negative regulation in the JAK/STAT pathway. In both cell lines, Apigenin reduces JAK2 and STAT3 phosphorylation, and in TF1 cells it also reduces STAT5 phosphorylation [112]. In the same study, Apigenin also impaired phosphorylation and activation of Src, an activator of STATs, in both cell lines [112]. Cao et al. (2016) showed the role of Apigenin in the regulation of STAT3 in melanoma B16F10 cell lung metastasis [113]. Apigenin enhances the immune response in these cells by inhibiting STAT3 and its direct target, VEGF, which has an immunosuppressive function. Apigenin also downregulates other STAT3 target genes like MMP2, MMP9, and TWIST1, thereby reducing cell migration and invasion [113].

The ethanolic extract of black raspberries has two abundant phytochemicals metabolites upon ingestion called Cyanidin-3-rutinoside and quercitin-3-rutinoside. Both reduced the phosphorylation of STAT3 in peripheral blood mononuclear cells treated with IL-6 [115]. In conclusion, flavonoids can reduce several inflammatory markers such as cytokines, chemokines, inflammatory enzymes, and proteins related to migration and invasion in cancer through modulation of NF-ĸB and JAK/STAT3 signaling, AP-1 complex, and NLRP3 inflammasome.

## 3. Flavonoids and Redox Metabolism

Reactive Oxygen Species (ROS) are among the most important mutagenic factors that occur naturally in the body and cause genetic instability within cells. Such instabilities can generate harmless or malignant mutations, which can eventually lead to cancer. It is known that malignant changes at the genomic level are the main pathological driving force of carcinogenesis, thus being frequently associated with oxidative stress [120]. Multiple biochemical reactions where oxygen is metabolized can lead to the generation of toxic reactive intermediates that can damage DNA [121]. Adaptive changes, which increase over long periods cumulatively, must occur within a cell for the malignant transformation. On the other hand, there are several examples of how genetic mutations (inherited or acquired) lead to increased ROS production, which in turn is associated with DNA damage and contributes to the malignant transformation [122,123]. Therefore, cancer cells depend on and adapt to this highly unstable and mutagenic environment, further highlighting the crucial role of oxidative stress in cancer [124].

The metabolic changes involved in carcinogenesis contribute to a high degree of oxidative stress in the tumor environment. However, oxidative stress defenses are adapted in cancer, enabling cancer cells to survive [124]. This is associated with the fact that most cancer cells no longer use the complete oxidative phosphorylation to generate ATP, but instead glycolysis, which is known as the Warburg effect. Such an effect has much influence not only on the energetics of cells but also on the redox system, the resilience, and the adaptation of cancer. Several molecular changes are known to relate the metabolic adaptations of cancer cells and the redox balance.

### 3.1. Metabolic Alterations in Cancer

In the Warburg effect, the M2 isoform of Pyruvate Kinase (PKM2), although less enzymatically efficient compared to the M1 isoform, is more commonly found in tumors due to its promotion via Myc Proto-Oncogene Protein C (c-Myc) [125]. The c-Myc oncoprotein affects the splicing of Pyruvate Kinase (PK) mRNA through the positive regulation of the Polypyrimidine Tract Binding protein (PTB) and heterogeneous nuclear Ribonucleoproteins (hnRNPs) A1 and A2, leading to the predominant production of PKM2 [126]. The less efficient PKM2 is advantageous for cell proliferation, as it enables the entry of carbohydrate metabolites from glycolysis into alternative pathways to produce macromolecules and Nicotinamide Adenine Dinucleotide Phosphate (NADPH), which are necessary for tumor growth and the support of altered redox balance [127]. This is accomplished since PKM2 shifts metabolic precursors from glycolysis to the pentose phosphate pathway to produce NADPH and ribose. Isocitrate Dehydrogenase Dependent on NADP 1 (IDH1), IDH2, and Malic Enzyme 1 (ME1) also contribute to the production of NADPH [128]. Myc increases glutamine uptake and glutaminolysis, leading to de novo synthesis of Glutathione (GSH). Therefore, Myc contributes to the production of NADPH, promoting the expression of PKM2. Together, NADPH and GSH control the increased levels of ROS driven by the increased proliferation of cancer cells [128].

With the influence of the Warburg effect, GSH plays a central role in the cellular antioxidant defense system in cancer. GSH participation in cellular metabolic redox processes and elimination of ROS is present in all existing aerobic organisms [129]. GSH is a non-enzymatic antioxidant that is intracellularly synthesized from cysteine, glycine, and glutamate and is highly abundant in all major cell compartments, such as cytosol, nuclei, and mitochondria [124]. At the cell nucleus, GSH protects sulfhydryl groups of proteins essential for DNA repair and gene expression. GSH antioxidant properties are also manifested in the direct elimination of hydroxyl radicals and singlet oxygen (O^2−^), hydrogen peroxide (H_2_O_2_), lipid peroxides, and even 4-Hydroxynonenal (HNE), in conjunction with the enzymatic action of Glutathione Peroxidase (GPx) and Glutathione Transferases (GSTs). GSH is also involved in the reductive regeneration of important antioxidants, including water-soluble vitamin C and lipid-soluble vitamin E [129]. The oxidized form of GSH is Glutathione Disulfide (GSSG), formed by the oxidation of two GSH molecules. GSSG is generally formed during the reduction of organic hydroperoxides and inorganic peroxides such as H_2_O_2_ in enzymatic reactions catalyzed by GPx or Peroxiredoxins (PRXs). GSSG can be reduced back to GSH in the tandem enzymatic action of Glutathione Reductase (GR) and the reducing equivalent NADPH + H^+^. Therefore, the GSH:GSSG ratio is considered an important indicator of redox balance in cells, where a higher ratio means less oxidative stress [130].

Along with GSH metabolism and the Warburg effect, there are enzymes and antioxidant factors that are largely modulated in cancer cells, such as Superoxide Dismutases (SODs), Catalase (CAT), NADP oxidases (NOXs), Nuclear Factor Erythroid 2-related Factor 2 (NRF2), COX-2, Nitric Oxide Synthase-2 (NOS2), and Hypoxia-Inducible Factor 1 alpha (HIF1α) [131,132,133,134]. HIF1α, which is also overexpressed in the Warburg effect, increases the expression of Glucose Transporters (GLUTs), glycolytic enzymes, and Pyruvate Dehydrogenase Kinase, isoenzyme 1 (PDK1), which blocks the entry of pyruvate into the Tricarboxylic Acid (TCA) cycle [128]. Myc cooperates with HIF1α for the activation of several genes that encode glycolytic proteins but also increases mitochondrial metabolism [135]. The cellular tumor antigen p53 opposes the glycolytic phenotype by suppressing glycolysis through TP53-Induced Glycolysis and Apoptosis Regulator (TIGAR), increasing mitochondrial metabolism via Protein SCO2 homolog (SCO2) [136,137]. Organic Cation Transporter 1 (OCT1) acts in the opposite way to activate the transcription of genes that drive glycolysis and suppress oxidative phosphorylation [138].

Another important factor for redox metabolism in cancer is NRF2, which is one of the main antioxidant transcription factors and regulates positively the expression of various antioxidant and detoxifying molecules [139]. When ROS levels are low, NRF2 binds to Kelch-like ECH-Associated Protein 1 (KEAP1), which triggers the degradation of NRF2. Under oxidative stress, p53 is activated and stimulates the expression of p21 [128]. p21 prevents KEAP1-NRF2 interaction and preserves NRF2, increasing antioxidant protection [139], through the nuclear heterodimerization with Musculoaponeurotic Fibrosarcoma Proteins (MAFs) [140]. The loss of p53 in a cancer cell inactivates this redox maintenance mechanism: as p21 is not activated, NRF2 continues to be degraded, antioxidant proteins are not expressed, and the redox balance is lost [128]. It may be possible to explore mutations of loss of p53 function or other tumor suppressor genes by applying additional oxidative stress. In the absence of redox maintenance pathways that are supported by these tumor suppressors, malignant cells can be selectively killed [141].

### 3.2. Flavonoids and Oxidative Stress in Cancer

The best-described property of almost all groups of flavonoids is their ability to act as antioxidants [142]. The antioxidant activity of flavonoids has already been shown to depend on the organization of functional groups on the nuclear structure of the molecule [7]. The configuration, substitution, and the total number of hydroxyl groups substantially influence the antioxidant activity, such as radical scavenging and metal ion chelation capacity [143]. The hydroxyl configuration of the B ring is the most significant determinant for the elimination of ROS and Reactive Nitrogen Species (RNS) because it donates hydrogen and electron to hydroxyl, peroxyl, and peroxynitrite radicals, stabilizing the flavonoid radical [7].

In healthy tissues, the antioxidant mechanisms of flavonoids may include suppression of ROS formation by inhibiting enzymes such as GST or by chelating trace elements involved in the generation of free radicals [144]; direct neutralization of ROS; and positive regulation or protection of antioxidant defenses [145]. In cancer, such mechanisms of action are also observed, however, the countless metabolic differences in cancer cells significantly affect the oxidative balance of the cell. Therefore, it is necessary to understand how the oxidative balance is affected in a cancer cell to understand the action of flavonoids in this context.

There is growing evidence of the importance of flavonoids in modulating the carcinogenic pathways associated with glucose metabolism that indirectly affect the redox balance of the cancer cell. Flavonoids target the activity of certain enzymes involved in aerobic glycolysis, the expression of transporters responsible for glucose uptake, the modulation of HIF1 in normoxic conditions, and several other factors associated with the Warburg phenotype [146].

**Figure 3:**
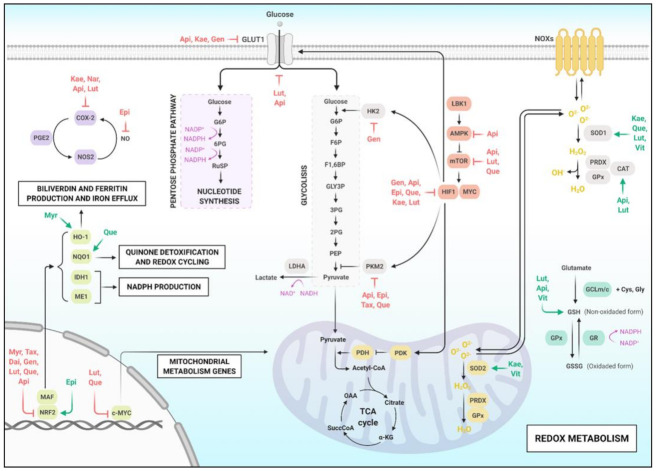
Flavonoids modulate redox metabolism pathways in cancer. Flavonoids, in addition to functioning as free radical scavengers, also act on antioxidant system enzymes such as Superoxide Dismutases (SODs), Catalase (CAT), Cyclooxygenase-2 (COX-2), Glutathione (GSH), and Glutathione Disulfide (GSSG) in cancer. Additionally, some flavonoids can interact with NRF2, activating many systems responsible for the redox balance and cell detoxification such as Heme Oxygenase-1 (HO-1), NAD(P)H Quinone Dehydrogenase 1 (NQO1), Isocitrate Dehydrogenase Dependent on NADP 1 (IDH1), and Malic Enzyme 1 (ME1). Hypoxia-Inducible Factor 1 (HIF1) has also been shown to be relocated, degraded, and inhibited by the action of flavonoids in cancer cells, thereby reducing receptors such as GLUT1 and glycolytic enzymes related to the Warburg effect such as PDK1, HK2, and PKM2, indirectly increasing the TCA cycle and not allowing the transformation of pyruvate into lactate. The inhibition of some cell growth proteins such as mTOR, AMPK, and AKT was also shown to be inhibited by flavonoids, affecting the c-Myc oncoprotein indirectly. Que: Quercetin; Epi: Epigallocatechin, Kae: Kaempferol; Myr: Myricetin; Que: Quercetin; Api: Apigenin; Gen: Genistein; Dai: Daidzein; Lut: Luteolin; Nar: Naringenin; Tax: Taxifolin; Eri: Eriodictyol; Vit: Vitexin. Original figure made for this review using the Biorender software.

Several inhibitory effects of flavonoids in cancer were analyzed in vitro through evaluations of the enzymatic activity of PKM2, as shown in Figure 3. Among these flavonoids, Taxifolin, Apigenin, Catechin gallate, and Epicatechin are the most effective in inhibiting PKM2 activity in in vitro experiments [147,148]. Additionally, Quercetin significantly decreased the level of glycolysis-related proteins, including PKM2, by modulating the protein kinase B (AKT) and mechanistic Target Of Rapamycin (mTOR) pathway in vivo [149]. Quercetin also reduced the level of PKM2 in the colon mucosa of F344 rats, pointing to a chemopreventive role of this flavonoid [150]. In the context of the Warburg effect, Kaempferol reduced the mRNA levels of the Glucose Transporter 1 (GLUT1) and prevented the uptake of Monocarboxylate Transporter 1 (MCT1), leading to the accumulation of extracellular lactate in breast cancer [151]. Epicatechin stimulated mitochondrial respiration in vitro in pancreatic cancer cells [152] and can act indirectly on the Warburg effect via MAPK/ERK2/Cytochrome c Oxidase (COX) [153]. In addition, flavonoids such as Icariside II, aspalatin, baicalein, and hesperetin are effective in important molecules in the Warburg effect, such as IDH1, IDH2, OCT1, PDK1, and NOX2 [154,155,156,157,158].

Flavonoids have shown direct effects on glutathione metabolism in cancer models. GSH and GST levels were increased after treatment with Luteolin in colorectal cancer models [159,160]. Apigenin treatment increased the expression of genes encoding phase II enzymes, blocking the NADPH oxidase complex and, consequently, downstream target inflammatory genes, which leads to increased NRF2 expression and nuclear translocation [161]. In addition to the proteins involved in cell growth and the Warburg effect, flavonoids have been shown to stabilize important oxidative factors such as HIF1 and NRF2, as already mentioned above [162].

The modulation of NRF2 expression for cancer therapy is well studied, both for cancer types that have it overexpressed, and for those that express it less. [163,164]. The class of NRF2-inducing compounds helps in the detoxification of carcinogens and environmental mutagens (Figure 3). They can also decrease the levels of ROS in tumor cells and make them susceptible to therapy [165]. Tamoxifen-resistant MCF7 breast cancer cells showed a response to a combination of EGCG and siRNA against NRF2 [166]. A similar mechanism has been observed in cervical cancer, where EGCG increased cisplatin activity and induced apoptosis [167]. Luteolin can potently inhibit NRF2 in A549 NSCLC cells, increasing their sensitivity to various anticancer drugs, as well as in a xenographic model of nude athymic mice [168]. Quercetin also directly interacts with NRF2: as part of the NAD(P)H Quinone Dehydrogenase 1 (NQO1) induction process, Quercetin binds to NRF2 protein and increases its half-life four times [169,170]. Apigenin inhibited the NRF2/ARE/CAT pathway in doxorubicin-resistant hepatocarcinoma (BEL-7402/ADM and HepG2) [171,172]. Apigenin inhibited NRF2, at the mRNA level, and detoxification enzymes in phase 2, at protein and mRNA levels, in BEL-7402/ADM cells [172], and sensitized doxorubicin-resistant (DOX-resistant) BEL-7402/ADM cells to doxorubicin (DOX), reducing the IC50 value of DOX treatment [173]. Myricetin activated NRF2 by regulating KEAP1 interaction, also decreasing NRF2 ubiquitination and increasing Heme Oxygenase-1 (HO-1) levels in hepatocarcinoma [174]. Epicatechin treatment has been shown to increase NRF2 phosphorylation and nuclear translocation also in hepatocarcinoma cell culture [175]. Taxifolin also inhibited TPA-induced colon carcinogenesis in albino Swiss mice through the epigenetic induction of NRF2 [66,176]. Genistein reduced the methylation level of the KEAP1 promoter region, leading to increased mRNA expression, thereby effectively inhibiting NRF2 transcription to the nucleus [177]. In this context, Vitexin increased p21 expression and decreased CDK1 in melanoma cell lines and a mice model, suppressing melanoma cell growth through DNA damage by increasing ROS levels [178].

Regarding HIF1, many flavonoids were also effective in the anticancer context, as shown in Figure 3. For example, Quercetin inhibited the accumulation of HIF1α, as well as its synthesis under hypoxia conditions and in a concentration-dependent manner in several cell lines, including LNCaP prostate cancer cells, SKBR3 breast cancer cells, and CX-1 colon cancer cells [179,180]. Interestingly, Quercetin increased the accumulation of HIF1α in healthy cells, showing improvement in the therapeutic index of DOX through its opposite effects on HIF1α in healthy and cancer cells [181]. EGCG also significantly suppressed the accumulation of HIF1α protein in gastric cancer cells but did not affect the expression of HIF-1α mRNA [182]. The mechanism linked to the HIF1 inhibitory properties of EGCG is explained by its interference with the PI3K/AKT/mTOR pathway and its translation function [183]. Luteolin suppressed HIF1 activation in M2-like tumor-associated macrophages under hypoxia conditions [184]. Kaempferol showed strong inhibitory effects on HIF1 activity in Huh7 hepatocellular carcinoma cells by relocating HIF1 into the cytoplasm due to inactivation of p44/p42 MAPK, which decreased cell viability under hypoxia conditions [185]. In addition to these flavonoids, Apigenin also downregulated hypoxia-responsive genes, such as HIF1α, GLUT-1, and VEGF, in human pancreatic cancer cells [186]. Genistein sensitized liver cancer cells to apoptosis, directly regulating HIF1α, inactivating GLUT1 and Hexokinase 2 (HK2) to suppress aerobic glycolysis [187].

When observing associated redox pathways, it is possible to identify several effects of flavonoids on cancer (Table 4). In vivo studies have indicated that Naringenin can suppress the early stage of colon cancer by attenuating levels of iNOS and COX-2 in mice injected with carcinogen [188,189]. Luteolin also inhibited iNOS and COX-2 [190]. The treatment of murine hepatomas with Daidzein and/or Genistein led to increased expression of Quinone Reductase (QR) mRNA and its activity, as well as greater NRF/ARE binding capacity [191]. Apigenin and Luteolin treatments in osteosarcoma models showed Forkhead box protein O1 (FOXO1) translocation and reduced Glucose-6-Phosphatase (G6Pc) mRNA levels, as well as Phosphoenolpyruvate Carboxykinase (PEPCK), CAT, and SOD [192,193]. Kaempferol triggered the generation of ROS and apoptosis by reducing the concentrations of thioredoxin and proinflammatory cytokines, also increasing SOD activity in glioblastoma cells [194]. On the other hand, in a colon cancer model, Kaempferol blocked the generation of ROS, causing the cell cycle to arrest in G1 and G2/M and affecting cell migration [195,196]. In stomach cancer, Kaempferol reduced the expression levels of COX-2, p-AKT, and p-ERK, which are involved in cell proliferation and cell cycle arrest [197]. Inflammation can also be inhibited by anthocyanins (Delphinidin, Cyanidin, and Petunidin) through the PI3K/AKT and NF-κB pathways, suppressing the expression of COX-2 and iNOS and regulating the expression of antioxidant enzymes from phase II via NFR2/ARE signaling [198,199]. Vitexin also decreased ROS levels and increased GSH and SOD levels in pheochromocytoma cells [200].

## 4. Flavonoids and Cell Growth Signaling

In mammalian cells, growth signaling is necessary from embryogenesis to adult cells, ensuring adequate functions in cells [203]. Hanahan and Weinberg described that one of the main and fundamental characteristics of cancer cells is to sustain a chronic proliferation state [14]. In normal cells, there is a fine regulation of the extracellular growth stimulus, while cancer cells develop metabolic autonomy to support chronic growth signaling [203]. Cancer cells enabling signals are based in large part on growth factors that bind cell-surface receptors, typically the Tyrosine Kinase Receptors (TKRs) [14]. These receptors are messengers of pathways that control tumor progression, including the AKT/mTOR and Ras/ERK signaling. In this topic we explore one of the hallmarks of cancer, focusing on what are the main targets of cell proliferation in which flavonoid compounds can act. A summary of the mechanisms of action of flavonoids in cell growth signaling is shown in Table 5.

### 4.1. The AKT/mTOR Pathway

**Figure 4:**
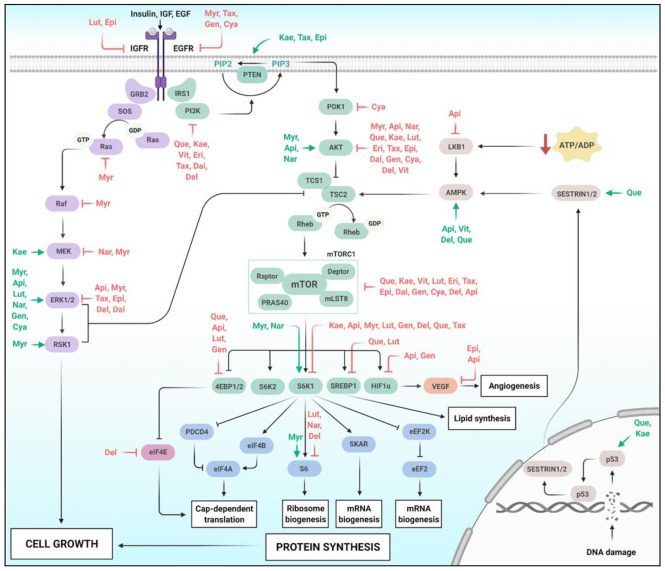
Flavonoids modulate growth pathways in cancer. Most of the flavonoid compounds inhibit the AKT/mTOR and Ras/ERK signaling pathway, impairing especially the metabolism, proliferation, survival, growth, and angiogenesis in cancer cells. Flavonoids can inhibit the response of the tyrosine kinase receptors (RTKs), initially controlling the upstream signaling cascade. The signaling can bifurcate in two main pathways: the Ras/ERK and the AKT/mTOR signaling. The Ras/ERK signaling, which is important in tumor progression, can be both negatively and positively regulated by flavonoids. In the AKT/mTOR pathway, one of the primary stages of signaling is the conversion of PIP2 to PIP3. This conversion can be inhibited by some flavonoids by inhibiting their converter, PI3K, or by stimulating the reverse conversion of PIP3 to PIP2 through PTEN, known as a tumor suppressor. Although some compounds stimulate AKT, probably through a feedback loop, most flavonoid compounds can inhibit AKT, preventing the activation of mTOR and, consequently, its effectors. The compounds directly inhibit mTOR, in addition to inhibiting 4EBP1/2 and S6K1, proteins that control especially the translation initiation and ribosomal and mRNA biogenesis. The compounds also stimulate p53, which targets SESTRIN 1/2, to inhibit the AKT/mTOR pathway by stimulating AMPK. mTOR signaling also controls lipid synthesis and angiogenesis, which can be both inhibited by flavonoids. These inhibitory regulations direct the cells to inhibition of cancer progression. Epi: Epigallocatechin, Kae: Kaempferol; Myr: Myricetin; Que: Quercetin; Api: Apigenin; Gen: Genistein; Dai: Daidzein; Lut: Luteolin; Nar: Naringenin; Tax: Taxifolin; Eri: Eriodictyol; Vit: Vitexin. Original figure made for this review using the Biorender software.

In tyrosine kinase signaling cascades, insulin and other growth factors, such as IGF, bind to their receptors to promote the recruitment of Insulin Receptor Substrate 1 (IRS1) and the production of Phosphatidylinositol (3,4,5)-triphosphate (PIP3) through the activation of Phosphoinositide-3-Kinase (PI3K) [204]. PI3Ks catalyzes the conversion of PIP2 to the second messenger PIP3 and its reaction is reversed by the Phosphatase and Tensin Homolog (PTEN) (Figure 4) [205]. The PIP2 conversion recruits and activates the AKT, which phosphorylates the Tuberous Sclerosis Complex 2 (TSC2), dissociating the TSC1/2 protein complex and promoting mTOR activation [204].

Most flavonoids can inhibit mTOR signaling early on. Vitexin [206,207], Taxifolin [208], and Eriodyctiol [209] decreased p-PI3K in human lung cancer cells, as Daidzein [210,211] in human breast and ovarian cancer cell lines, and Delphinidin [212] in human lung cancer cells. Some of these compounds decreased p-PI3K while increasing PTEN expression, such as Kaempferol in hepatocarcinoma [213] and cervical cancer cells [214] and EGCG in pancreatic cancer cells [215], reinforcing the inhibition of mTOR signaling by these two axis. Remarkably, Taxifolin can bind to EGFR and PI3K and decrease their activities in murine epidermal JB6 P+ cells [216].

mTOR has the same catalytic subunits in two distinct complexes (mTORC1 and 2), which respond differently to rapamycin: while complex 1 has most functions inhibited, complex 2 is insensitive to the acute treatment [217]. Since its discovery, the mTOR pathway has been suggested as a central mechanism that converges different signaling pathways to coordinate cell growth [218]. Thus, mTOR acts as a sensor of energy, nutrient availability, and growth factors to orchestrate assertively catabolism and anabolism [218]. Overactivation of mTOR is commonly reported in cancer and is extensively associated with poor prognosis [219,220]. The sensitivity to mTOR inhibition by rapamycin may vary by several orders of exposure, indicating an intrinsic resistance to mTOR inhibition in different cancer types [221].

When active, mTORC1 phosphorylates substrates that control the production of proteins, lipids, nucleotides, and ATP, initiating anabolic programming while limiting the autophagic process [218]. During protein synthesis and translation initiation, mTOR mainly phosphorylates Eukaryotic Initiation Factor 4E-Binding Proteins (4EBPs) and p70 S6 Kinase 1 (S6K1), leading to the release of Eukaryotic Translation Initiation Factor 4E (eIF4E) by 4E-BP1 and Ribosomal Protein S6 (S6/RPS6) phosphorylation by S6K1 (Figure 4) [218,222,223]. The flavonoids Quercetin [224], Apigenin [225], and Luteolin [226] decreased 4E-BP1 phosphorylation in breast, keratinocytes, and lung cancer cells, respectively, presumably limiting protein translation. Notably, Quercetin at 15 µM decreased p-4EBP1, p-S6K1, p-AKT in breast cancer cell culture and decreased tumor growth after 13 weeks of treatment in the MDA-MB-231 xenograft model, highlighting that flavonoid as a rationally approach against breast tumor progression [224].

Since cell growth demands lipid synthesis, mTOR also coordinates the lipid synthesis through two main processes: activation of Sterol Regulatory Element-Binding Proteins 1/2 (SREBP1 and 2) and Peroxisome Proliferator-Activated Receptor-γ (PPARγ) (Figure 4) [227]. Luteolin significantly reduced the mRNA levels of SREBP1 and SREBP2, decreasing SREBP1 protein expression in JAR and JEG-3 Human Placental Choriocarcinoma Cells [228]. Furthermore, in C6 rat glioma cells, Quercetin inhibited cholesterol and fatty acid synthesis, decreasing the expression of SREBP1, SREBP2, and ChREBP, a transcription factor that regulates genes involved in lipogenesis [229].

AKT, an oncogene, is highly expressed in human cancers and can be considered the central and convergent point of several growth signaling pathways, including mTOR [230]. AKT is capable of phosphorylating several downstream effectors, such as apoptotic proteins, transcription factors, and other oncogenes [231]. Most of the compounds with anti-cancer activity decrease AKT activation as the main molecular mode of action. Quercetin suppressed breast cancer progression by decreasing AKT/mTOR pathway, inducing autophagy, in vitro and in vivo [149], and inhibited AKT-mediated activation of mTOR and its effectors in hepatocellular carcinoma [232], prostate [233], and breast cancer cells [234]. Similarly, in concentrations ranging between 25–100 µM, Kaempferol decreased AKT phosphorylation in hepatocarcinoma [213], leukemia [235], endometrial cancer [236], renal carcinoma [237], and human lung cancer cells [238].

Despite AKT/mTOR inhibition being an important strategy for cancer treatment, some mTOR inhibitors, such as ‘rapalogs’, presented a feedback loop activation of the AKT/mTOR axis, becoming cytostatic rather than cytotoxic [218]. Natural compounds inhibit the mTOR effectors 4E-BP1, S6K1, and S6, along with AKT inhibition [239]. In human breast cancer cells, Genistein and Daidzein potentially inhibited cell migration and invasion while decreasing PI3K and AKT phosphorylation [240]. Decreased PI3K, AKT, and mTOR phosphorylation were also observed in human breast cancer cells at 50 µM for 48 h [210]. Remarkably, Myricetin [241,242], Apigenin [243,244] and its derivative Vitexin [206,207], Luteolin [226,228,245], Eriotyctiol [62,209], Taxifolin [216,246,247], Naringenin [248,249], EGCG [215,250,251], and Cyanidin [252,253,254] also decreased AKT phosphorylation and mTOR effectors in several cancer models, avoiding this feedback activation loop.

Regarding AKT, all studies presented here involving Genistein [255,256,257,258,259] and Delphinidin [212,260,261,262,263] treatment decreased AKT activation, highlighting that as a robust effect of these two flavonoids in different types of cancer. The co-treatment with indol-3-carbinol, a compound extracted from Cruciferous vegetables, and Genistein increased subG1 cell accumulation and significantly decreased p-AKT, while inhibiting p-mTOR in HT-29 human colon cancer cell line [255]. Genistein also sensitized HeLa cervical cancer cell line to cisplatin, while preventing the increase in p-mTOR, p-AKT, and p-S6K1 generated by cisplatin treatment [256]. At 80 µM, Delphinidin substantially decreased p-mTOR, p-AKT, and p-eIF4E and increased p-LKB1 and p-AMPK, resulting in the induction of autophagy in MDA-MB-453 and BT474 cells [260].

### 4.2. The Ras/ERK Pathway

In addition to AKT, one of the most important pathways that control cell growth is the Ras/ERK signaling pathway, responsible for alterations in cell morphology, differentiation, and neoplastic transformation [264]. The gain-of-function mutations in members of the Rat Sarcoma viruses (Ras) family are one of the most common genetic alterations in cancer [265], which leads to prolonged activation of Ras signaling [264]. The first Ras effector pathway identified was the RAF/MEK/ERK axis and the second best-characterized Ras effector is PI3K, cross-talking with the mTOR/AKT pathway (Figure 4) [265,266]. The first evidence of the interaction between Ras and PI3K was achieved by detecting PI3K activity in Ras immunoprecipitation in transformed cells [267].

Different strategies that can inhibit these pathways are widely considered therapeutic interventions in cancer [268]. Flavonoids appear to synergistically inhibit both Ras and mTOR signaling, such as Apigenin in melanoma cell lines [244], Daidzein [269] in human prostate cancer cell lines, Delphinidin [212,263] in lung and ovarian cancer cells, and EGCG in colorectal cells in vitro and in vivo [250]. Studies reported that Myricetin can decrease both AKT and ERK 1 and 2 phosphorylation (also known as Mitogen-activated protein kinase 2, MAPK2, and Mitogen-Activated Protein Kinase 1, MAPK1, respectively) [135,242,270,271]. Myricetin can reduce the phosphorylation of AKT while decreasing the activation of Ras, RAF proto-oncogene serine/threonine-protein kinase (Raf), and ERK in human glioblastoma cells [242]. Myricetin also showed to be a potent Mitogen-Activated Protein Kinase Kinase 1 (MEK1) inhibitor, leading to decreased ERK phosphorylation and, consequently, impairing the neoplastic transformation of mouse epidermal cell lines [135]. Thus, flavonoids are highlighted as important compounds in adjuvant cancer therapy, presenting potent inhibition activity against cancer cell growth and proliferation.

## 5. Flavonoids and Autophagy

Autophagy is a cell survival pathway that is composed of 3 routes: macroautophagy, microautophagy, and chaperone-mediated autophagy. Macroautophagy is the most studied one and a catabolic process, responsible for the degradation of organelles within a vesicle derived from the smooth endoplasmatic reticulum and mediated by lysosomal hydrolytic enzymes. This pathway is activated to ensure the maintenance of homeostasis in cases of starvation, dysfunctional organelles, and stress compounds such as ROS inducers. Macroautophagy is also modulated in cases of cell proliferation, differentiation and directly interacts with the apoptotic pathway [281].

**Figure 5:**
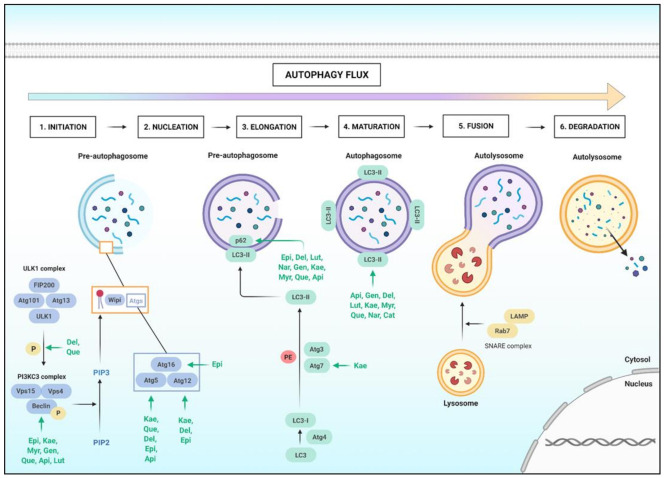
Flavonoids modulate autophagy pathways in cancer. Macroautophagy begins by activation of ULK complex in the endoplasmic reticulum that leads to Beclin1 phosphorylation this permits the activation of PI3KC3 complex that converts PIP2 to PIP3 promoting an accumulation of phospholipid, this accumulation recruits Wipi proteins and some autophagy-related genes (Atg 5,12 and 16) for the formation of the single-layer pre-autophagosome (in blue), the autophagy receptor p62 interacts with the autophagy target and binds to active LC3-II forming a mature double membrane (in purple) that is fused with lysosomes that will digest the autophagosome content with cathepsin enzyme. Flavonoids can stimulate macroautophagy, through the increase in expression of Beclin1, p62, LC3-II, and some Atgs. Kae: Kaempferol, Myr: Myricetin, Que: Quercetin, Api: Apigenin, Gen: Genistein, Del: Delphinidin, Lut: Luteolin, Nar: Naringin, Epi: Epigallocatechin. Original figure made for this review using the Biorender software.

As shown in Figure 5, the macroautophagy pathway starts by the formation of the Unc-51 Like Autophagy Activating Kinase (ULK1) complex, that activates by phosphorylation the Beclin1 complex, a type of Phosphoinositide 3 Kinase 3 Complex (PI3K3C), leading to the production of Phosphoinositol 3 Phosphate (PIP3) from Phosphoinositol 2 Phosphate (PIP2). The PIP3 accumulation recruits WD-repeat protein Interacting with Phosphoinositides (Wipi) and some autophagy-related genes (Atgs) proteins for the formation of a single-layer pre-autophagosome. In the next step, there is the nucleation of the pre-autophagosome, generating a double layer membrane. This occurs when the Microtubule-associated protein 1A/1B-light chain 3 (LC3-I) protein is lipidated to the form LC3-II, binding to the autophagosome by autophagy receptors such as sequestosome-1 (p62). Lastly, the lysosomes fuse with the autophagosome, generating the autolysosome, which digest the content by Cathepsins enzymes action [282,283,284].

Currently, the macroautophagy pathway has been intensively studied in oncology, since it is related to some important pathways that frequently present mutations in tumoral models, related to proliferation (mTOR and ERK) and survival and DNA repair (Bcl-2, p53). In addition, the dysregulation of autophagy is related to some of the hallmarks proposed by Weinberg and Hanahan in 2011 [14,285], and other relevant characteristics to tumors as presented below:

- Autophagy and genome instability and mutation: autophagy acts as a clearance pathway that removes dysfunctional organelles. These organelles can lead to the accumulation of toxic compounds, such as ROS, that increase genomic instability, promoting mutations [286,287].
- Autophagy and sustaining proliferative signaling: the regulation of this process by some molecules such as the accumulation of Adenosine Monophosphate (AMP) and the depletion of Adenosine Triphosphate (ATP) leads to the activation of anti-proliferative pathways, for example, the Liver Kinase B1 (LKB1) and AMP-activated Kinase (AMPK) pathways. These proteins reduce the rate of cell proliferation and also intensify the autophagy process. In some types of cancer with driver mutation in PTEN and LKB1, there is a decrease of autophagy to maintain high levels of proliferation [288,289].
- Autophagy and deregulation of cellular energetics: some types of cancer promote reprogramming of cellular bioenergetics known as the Warburg effect, where autophagy plays a critical role to protect the cell against high levels of ROS and lactate, helping cell survival [290,291].
- Autophagy and resistance to cell death: Beclin1 protein interacts with Bcl-2 protein, inhibiting the autophagy process. Thus, when a cancer cell receives a death stimulus such as chemotherapy, the interaction between Beclin1 and Bcl-2 proteins is lost and the cell presents high levels of anti-apoptotic and autophagy activities, turning it resistant to apoptosis [292,293].
- Autophagy and induction angiogenesis: when the supply of O_2_ and nutrients are low, autophagy plays an important role in cell survival. However, some types of cancer can purposefully decrease autophagy pathways to promote tumoral angiogenesis [294,295].
- Autophagy and activation of invasion and metastasis: low levels of autophagy are related to EMT with the association of inflammation and macrophages in the tumor. Cancer cells can then detach from the primary tumor region, promoting the metastasis process and producing an invasive tumoral colony. Tumoral colonies can benefit from autophagy since they can avoid the anoikis process, a type of apoptosis due to the loss of contact from the extracellular matrix. Autophagy also plays an important role to overcome starvation and hypoxia until the metastatic cells invade a new region [296,297].
- Autophagy and chemoresistance: some types of cancer have mutations on proliferative pathways such as p53 and MAPK and are known as “autophagy addicted”. The scenario of intense proliferation can be associated with a cellular metabolic disturbance due to the preference for glycolytic metabolism. In this case, autophagy plays a crucial role in tumor progression, protecting the cell against the damage of super proliferation and generating energy substrates through the degradation of organelles and proteins. Thereby, when the autophagy pathway is activated, the cells often present anti-apoptotic activity caused by Beclin1 interaction with Bcl-2, through the BH3 domain, which can lead to chemotherapeutic resistance [292,298].
- Autophagy and senescence: evidence points out that autophagy may correlate with the senescence process. CDKs (Cyclin-dependent-kinase) and cyclins control important pathways in proliferation, such as mTOR and AMPK, that can regulate autophagy. Thus, the deregulation of this system can slow the senescence process [299,300].

In this sense, it is important to highlight the autophagy process in oncology studies. According to some authors, autophagy inhibitors such as chloroquine, 3-Methyladenine (3-MA), and bafilomycin can increase the cancer sensitivity to chemotherapy and can be associated with therapies [301]. Other ways to modulate the autophagy process in cancer studies were demonstrated by using natural compounds. The literature points to strong evidence of the potential of flavonoids in stimulating autophagy in cancer. Table 6 describes the flavonoids that present solid evidence regarding the modulation of the autophagy process. Studies describe Quercetin, Apigenin, Luteolin, Kaempferol, Myricetin, Delphinidin, Naringin, Catechin, and Genistein as flavonoids targeting autophagy [32].

A group of flavonoids showed great potential in stimulating the formation of autophagosomes through the increase in expression of ULK1, PIP3K3C complex, some Atgs, p62, and LC3-II in different types of cancer. In breast, liver, and prostate tumor models, Quercetin specifically increased Atg5, Beclin1, p62, and LC3-II protein contents [149,302,303,304]. Kaempferol in liver and colon tumor models increased Atgs 5, 7, 12, Beclin1, p62, and LC3-II protein contents [305,306,307,308]. Myricetin in breast, melanoma, and colorectal tumor model increased Beclin1, p62, and LC3-II protein contents [309,310,311]. Flavones can also modulate autophagy. Apigenin in breast, glioma, and liver tumor models modulated autophagy and autophagosome development through the increase of Atg5, Beclin1, p62, and LC3-II [302,312,313,314,315]. Luteolin in liver and squamous tumor models modulated autophagy by increasing Beclin1, p62, and LC3-II [316,317,318].

Flavanones group also showed effects of stimulating the autophagy pathway. Naringin in gastric tumor models presented great potential for increasing Beclin1 and LC3-II protein content, stimulating the formation of autophagosome [319]. Anthocyanins such as Delphinidin in breast, osteosarcoma, and lung tumor models increased p-ULK, Atg5, 12, p62, and LC3-II protein content, indicating the stimulus of autophagosomes formation [260,320,321]. Epigallocatechins of the Flavans group in melanoma, colorectal, lung, and oral tumor models showed the ability to increase Atgs 5, 7, 12, 16, Beclin1, p62, LC3-II protein content [322,323,324,325,326]. Finally, isoflavonoids are indicated as great stimulators of the autophagy pathway, such as Genistein in breast, lung, and pancreatic tumor models, by increasing Beclin1, p62, and LC3-II protein content [327,328,329,330,331].

Currently, some authors have reported that bioactive compounds such as flavonoids present strong anticancer properties associated with chemotherapy treatment in cancer models [313,332]. Flavonoids, as already mentioned, can increase the canonical autophagy pathway and modulate non-canonical autophagy pathways, through the inhibition of proliferative routes (mTOR, Ras), hypoxia, and cytokines routes (STAT3). In addition, flavonoids may increase toxicity when associated with chemotherapeutic treatment. Ultimately this overactivation of autophagy may lead cells to a cytotoxic autophagy process, which is known as autophagic cell death or type II cell death [333].

Finally, it is worth mentioning the duality of autophagy in cancer. Firstly, this pathway can act to prevent cancer, through the removal of dysfunctional organelles, decrease of inflammation, removal of toxic compounds such as ROS, protection of DNA against damage and mutations, and maintenance of homeostasis. However, the autophagy pathway can be purposely modulated by cancer cells for the acquisition of hallmarks, becoming more aggressive and resistant to therapies and leading to a worse prognosis of the disease. Thus, the autophagy pathway can support tumorigenesis and its modulation may be carefully considered for cancer treatment [334,335].

## 6. Flavonoids, Apoptosis, and Cell Cycle

Apoptosis, a type of programmed cell death, occurs during early development and, in adults, it is used to eliminate from the body cells that have been damaged beyond repair. For this reason, apoptosis also plays an important role in preventing cancer [336]. Cells under apoptosis present a series of distinct changes in their morphology, such as loss of cell attachment, cytoplasmic contraction, and DNA fragmentation. In addition, a class of proteins is recruited, called caspases, which can be activated through the extrinsic or intrinsic apoptotic pathways [337].

**Figure 6:**
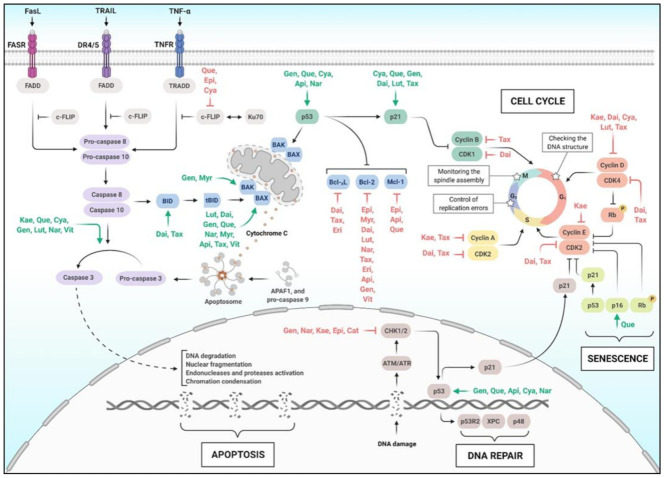
Flavonoids modulate apoptosis, cell cycle, DNA repair, and senescence pathways in cancer. Flavonoids can promote both down-regulation of anti-apoptotic proteins such as Bcl-2, Mcl-1, and Bcl-xL or upregulation of pro-apoptotic proteins as BAK, BAX, and BID. Besides that, flavonoids can also induce the conversion of pro-caspase 3 to activated Caspase 3. All these modulations lead to cancer cell death. Flavonoids also regulate proteins involved with the cell cycle, as p21, CDK1, CDK2, CKD4, and cyclins: D1, A, B, and E, culminating in cell cycle arrest. Proteins involved with DNA damage response as CHK1/2 also are modulated by flavonoids, inhibiting the ability of cancer cells to repair their DNA directing them to apoptosis. Quercetin can also regulate p16, a senescence marker. Epi: Epigallocatechin; Cat: Catechin; Kae: Kaempferol; Myr: Myricetin; Que: Quercetin; Api: Apigenin; Gen: Genistein; Dai: Daidzein; Cya: Cyanidin; Vit: Vitexin; Lut: Luteolin; Nar: Naringenin; Tax: Taxifolin; Eri: Eriodictyol. Original figure made for this review using the Biorender software.

Intrinsic apoptosis occurs inside the mitochondria, where its outer membrane is permeabilized and leads to cytochrome c release into the cytoplasm. The cytochrome c releasing is stimulated by pro-apoptotic proteins such as Bcl-2-Like Protein 4 (BAK) and Bcl-2 Homologous Antagonist/killer (BAX), leading to caspase 3 activation and apoptosome formation. Bcl-_X_L, Bcl-2, and Induced Myeloid Leukemia Cell Differentiation Protein (Mcl-1) are proteins of the intrinsic apoptosis pathway that can be inhibited (Figure 6) [338].

Extrinsic apoptosis, however, occurs outside the mitochondria and starts with the death receptors, which are cell membrane receptors known as Fas Receptor (FASR), Death Receptor 4/5 (DR4/5), and TNFR (Figure 6). Upon ligand binding, Fas Ligand (FasL), TNF-Related Apoptosis-Inducing Ligand (TRAIL), and TNF-α, respectively, the death receptors trimerize and recruit adaptor proteins, such as Fas-Associated Death Domain (FADD) and TNFR-associated Death Domain (TRADD), and initiator caspases (caspase 8 and 10) (Figure 6) [339].

### 6.1. Apoptotic Pathway

In the cancer context, apoptosis is critical, and the regulation of pro-apoptotic and anti-apoptotic proteins is well studied. Among the modulators of the apoptotic pathway are the flavonoids, which have been shown to activate pro-apoptotic processes in cancer cell lines and animal models [340]. A summary of these actions of flavonoids in apoptosis is described in Table 7.

In pancreatic cancer cells, Kaempferol upregulated caspase-3, which is a pro-apoptotic protein [341]. The same was observed for Quercetin and Cyanidins in cervical cancer cells [55,342], Genistein in colon cancer cells (HT29) [343], Luteolin in breast cancer cells (MDA-MB-231) [344], Vitexin in renal cancer cells [206], and Naringenin in hepatocellular carcinoma (HepG2) (Figure 6) [345].

Other pro-apoptotic proteins were described as upregulated by flavonoids, such as Caspase 9, BAK, BH3 Interacting-domain Death Agonist (BID), and BAX. Increased levels of BAX, for example, were observed in cancer cells treated with Myricetin, Vitexin, Quercetin, Apigenin, Genistein, Daidzein, Luteolin, Naringenin, and Taxifolin (Figure 6) [55,206,344,345,346,347,348,349,350]. The effects of flavonoids in pro-apoptotic proteins were not only detected in cancer cell cultures but also in athymic nude mice models, where 23 days of treatment with 0.2–0.4 mg/kg of Genistein was able to significantly increase the levels of BAX and BAK [348]. The downregulation of anti-apoptotic proteins has also been observed in the literature. The levels of Bcl-2 were lower in renal carcinoma, human colon cancer, prostate cancer, human leukemia, and hepatocellular carcinoma cells treated with Epigallocatechin, Myricetin, Apigenin, Genistein, Daidzein, respectively, compared with control cells (Figure 6) [346,347,348,351,352].

p53, a key protein related to several pathways involved in cancer, is also found modulated by flavonoids. In several types of cancer, p53 is mutated, losing its function and becoming inactivated [137]. When inactivated, apoptosis is blocked and the cell cycle is facilitated, leading to the uncontrolled proliferation and growth of cells. In leukemia cells (Nalm6) treated with 20 µM of Quercetin for 24 h, the total content of p53 was significantly increased [353]. The same occurred in ATL cells from adult T-cell leukemia treated with 50–100 µM of Apigenin for 16 h [354] and in HeLa cells treated with Cyanidins for 24 h (Figure 6) [342]. These collected data strongly support the evidence of the influence of flavonoids on cancer cell apoptosis, acting as isolated treatments or combined with other therapies.

### 6.2. Cell Cycle Control

The cell presents a series of events, in a cycle, that prepare the cell for its division and duplication to produce daughter cells [337]. The cell cycle has four steps–named G1, S, G2, and M–in which the cell increases in size, duplicates its genetic material, prepares for division, and divides, respectively. Each step is ordered and programmed [355]. Several molecules stimulate the cell cycle, including the cyclins, proteins that promote the activation and binding to Cyclin Dependable Kinases (CDKs) (Table 7), both working as protein complexes that orchestrate the progression and maintenance of the cell cycle. On the other hand, there are specific points in the cell cycle, called checkpoints, that stop the cell cycle to analyze the integrity of DNA, aiming at the production of daughter cells with correct genetic material [356]. In several types of cancer, the fine control of these checkpoints is lost, and cell proliferation is unlimited. For this reason, the search for molecules that promote cell cycle arrest is a promising therapeutic approach against cancer [357].

Flavonoids regulate cell growth and cell cycle progression by modulating the expression of these cell cycle regulatory proteins (Cyclins and CDKs) [337]. For instance, the treatment with Kaempferol downregulated the expression of cyclin D, E, and A in Human colon cancer cells (HT-29) [196]. Other flavonoids have been found to downregulate cyclins such as Daidzein, Cyanidin, Luteolin, and Taxifolin (Figure 6) [196,342,344,358].

Some flavonoids have been reported to mediate G2/M cell cycle arrest via the upregulation of the expression of Cyclin-Dependent Kinase Inhibitor 1 (p21). p21 is a protein that is activated by p53 and promotes the inhibition of cyclin B and E. HeLa cells, derived from cervical cancer, presented lower p21 expression when treated with Quercetin for 24 h [55]. The same was observed in a human colon cancer cell (HCT116) treated with Genistein [359], breast cancer cells (MDA-MB-231) under 10–30 µM of Luteolin [344], and colorectal cancer (HCT116) treated with Taxifolin for 24 h [350].

### 6.3. DNA Damage and Repair

The DNA is constantly damaged by exogenous and endogenous sources such as genotoxic chemicals, ultraviolet (UV), ionizing radiation, and reactive oxygen and nitrogen species [360]. The resulted damage in DNA can be double or single-strand breaks, base oxidation, deamination, interstrand crosslinks, or adduct formation. For each of these damages, there are specific cascades of molecules able to repair the DNA and to protect the cells from replicating with wrong genetic information [360]. DNA damage is one of the major causes for cancer initiation and progression due to genetic alterations, which may lead to loss of tumor suppression or increase of oncogenes [360].

Cancer treatments based on this pathway aim to decrease the expression of proteins that stimulate DNA repair, leading the cells to death when this treatment is combined with chemotherapy that induces DNA damage. Kuo et al. (2016) treated breast cancer cells MDA-MB-231 with flavonoids and observed decreased DNA repair pathway [360]. These cells were submitted to UV radiation and treated with different concentrations of Catechin and Epigallocatechin. The treatments with 5–10 µM of Catechin and 10–40 µM of Epigallocatechin were able to significantly reduce the phosphorylation of Checkpoint Kinase 1 (CHK1), a protein involved in DNA repair signaling. The same study submitted MDA-MB-231 cells to pretreatment with 10 μM or 50 μM of flavonoids followed by exposure to 10 μM cisplatin for 6 h to induce CHK1 and Checkpoint Kinase 2 (CHK2) phosphorylation. At 50 µM the flavonoids Kaempferol, Genistein, Naringenin, and Epigallocatechin were effective to reduce the levels of CHK1 and CHK2 phosphorylated (Figure 6) [360].

### 6.4. Cell Senescence

Cellular senescence is described as the irreversible arrest of cell proliferation and this phenomenon can be observed in several types of cells. The senescence occurs due to the gradual loss of telomere length after each cell division, and for this reason, this process is closely involved with the aging of cells [361]. As senescence leads to cell cycle arrest, at least in part, this process is linked to cancer suppression, being regulated by two major pathways: the p53/p21; and the cyclin-dependent kinase inhibitor 2A and Retinoblastoma-associated protein (p16INK1a/pRB), which are considered senescence markers [362]. Indeed, cells with mutations in p53 or p16INK4a present the inability to respond to certain senescence-inducing signals, which greatly increase cancer susceptibility [363].

Besides the inability to proliferate, the senescent cells have the feature of secreting several proinflammatory cytokines, chemokines, growth factors, and proteases, a process termed senescence-associated secretory phenotype (SASP) [362]. Although the SASP cells can attract immune cells to promote the clearance of the tumor, these senescent cells are also able to create a microenvironment that stimulates the cancer cells growth and metastasis, including the release of Stromelysin3 (MMP3) and VEGF, which are cancer inductors of metastasis and angiogenesis, respectively [361].

Studies already reported the effect of flavonoids in eliminating senescence cells (a process called senolytic effect) and consequently impairing the tumor progression [364]. Pro-senescence therapy was also recently proposed as an anti-cancer strategy and has been shown to effectively inhibit cancer. Özsoy et al. (2020) treated colon adenocarcinoma cell lines (Colo-320 and Colo-741) with Quercetin for 48h and showed a higher immunoreactivity to p16 [365]. The treatment with ECGC was able to induce senescence in U251 human glioblastoma cells, culminating in the shortening of telomere and cell cycle arrest [366]. Lastly, Banerjee et al. (2015) showed the effectiveness of apigenin in improving the activity of β-galactosidase in HCT-15 cells, leading the cell to senescence [367] (Figure 6).

## 7. Conclusions

In conclusion, flavonoids are important natural compounds with broad effects described in several signaling pathways related to cancer. Flavonoids can inhibit inflammation and increase immunity, modulating the NF-κB, MAPK, inflammasome, and JAK-STAT pathways, which are usually altered in cancer. In addition, flavonoids inhibit growth signaling pathways, such as the mTOR and Ras oncogenic pathways, and, at the same time, activate apoptosis and cause cell cycle arrest. In the case of autophagy and oxidative stress, which flavonoids can also modulate, both have a dual relationship with cancer depending on the stage of the disease. Thus, future studies must cautiously address these pathways in this context. Although more studies are needed to explore details on how flavonoids exert their mechanism of action in cells, the increasing amount of evidence strongly suggests the potential use of flavonoids as anti-cancer bioactive compounds.

## Figures and Tables

**Table 1:** Chemical structures, subclasses, and food sources of flavonoids.

Chemical Structure 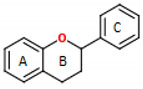	Subclass	Examples	Food Source
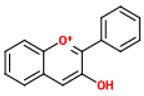	Anthocyanidins	Cyanidin (Cya), Delphinidin (Del)	Strawberry, Blackberry, Grape, Red cabbage [8]
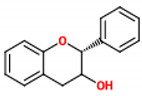	Flavanols	Catechin (Cat), Epigallocatechin (Epi)	Tea, Apples, Cocoa [9]
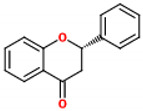	Flavanones	Naringenin (Nar), Taxifolin (Tax), Eriodictyol (Eri)	Citrus fruits [10]
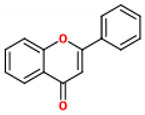	Flavones	Luteolin (Lut), Apigenin (Api), Vitexin (Vit)	Celery, Parsley, Artichokes, Chicory, Tea and Herbs leaves [11]
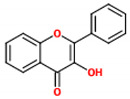	Flavonols	Quercetin (Que), Kaempferol (Kae), Myricetin (Myr)	Broccoli, Garlic, Onion [12]
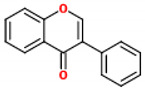	Isoflavones	Genistein (Gen), Daidzein (Dai)	Soy [13]

**Table 2:** Clinical trials based on flavonoids administration for cancer.

Anthocyanidins						
	Indication	Number of Participants	FDA Approval Status	Trial Status	National Clinical Trial Number (NCT#)	Ref.
Cyanidin	Breast Cancer	300	Not applicable	Completed	NCT02195960	-
	Colorectal Adenocarcinoma	100	Phase II	Unknown	NCT01948661	-
	Myelodysplastic Syndrome/ Myeloproliferative Neoplasm	21	Phase II	Active, not recruiting	NCT03140280	-
	Oral Cancer	58	Not Applicable	Not yet recruiting	NCT04372914	-
**Flavanols**						
	**Indication**	**Number of Participants**	**FDA Approval Status**	**Trial Status**	**NCT#**	**Ref.**
Catechins	Prostate Cancer	50	Phase I	Completed	NCT00459407	[19]
	Breast Cancer	40	Phase I	Completed	NCT00516243	[20]
	Breast Cancer	1075	Phase II	Completed	NCT00917735	[21]
	Lung Cancer	53	Phase II	Completed	NCT00573885	-
	Unspecified Adult Solid Tumor,	Unspecified	Phase I	Completed	NCT00091325	-
	Esophageal Cancer	55	Phase I	Completed	NCT00233935	-
	Cervical Cancer	98	Phase II	Completed	NCT00303823	[22]
	Bladder	31	Phase II	Completed	NCT00666562	-
**Flavanones**						
	**Indication**	**Number of Participants**	**FDA Approval Status**	**Trial Status**	**NCT#**	**Ref.**
Hesperidin	Breast Cancer	40	-	Completed	NCT03482401	[23]
**Flavones**						
	**Indication**	**Number of Participants**	**FDA Approval Status**	**Trial Status**	**NCT#**	**Ref.**
Apigenin	Colorectal Cancer	382	Phase II	Suspended	NCT00609310	-
Luteolin	Tongue Neoplasms	4	Early phase I	Unknown	NCT03288298	-
**Flavonols**						
	**Indication**	**Number of Participants**	**FDA Approval Status**	**Trial Status**	**NCT#**	**Ref.**
Quercetin	Prostate Cancer	31	Phase I	Active, not recruiting	NCT01912820	-
	Prostate Cancer	60	Not applicable	Recruiting	NCT01538316	-
	Squamous Cell Carcinoma	55	Phase II	Recruiting	NCT03476330	-
Quercetin-3-O-glucoside	Colorectal and Pancreatic Cancer	64	Phase II/III	Active, not recruiting	NCT02195232	-
	Renal Cell Carcinoma and Kidney Cancer	104	Phase I/II	Recruiting	NCT02446795	-
**Isoflavones**						
	**Indication**	**Number of Participants**	**FDA Approval Status**	**Trial Status**	**NCT#**	**Ref.**
Genistein	Bladder Cancer	60	Phase II	Active, not recruiting	NCT00118040	[24]
	Colon and Rectal Cancer	13	Phase I/II	Completed	NCT01985763	[25]
	Refractory Leukemias, Central Nervous System Tumor, Solid Tumor, Refractory Solid Tumor, Leukemia, and Lymphoma	6	Phase I/II	Completed	NCT02499861	-
	Non-small Cell Lung Cancer	21	Phase I/II	Active, not recruiting	NCT02567799	[26]
	Adenocarcinoma	44	Phase I/II	Unknown	NCT01182246	-
	Cancer	15	Phase I	Completed	NCT00001696	-
	Prostate Cancer	24	Phase II	Temporarily suspended	NCT02766478	-
	Prostatic Neoplasms	47	Phase II	Unknown	NCT00546039	-
	Breast Cancer	126	Phase II	Completed	NCT00290758	-
	Prostate Cancer	15	Phase II	Completed	NCT01325311	-
Daidzein	Prostate Cancer	43	Phase II	Completed	NCT00669656	-

**Table 3:** Molecular mechanisms of flavonoids in inflammation, immunity, and cancer.

Inflammation and Immunity						
	Molecular Mechanisms	Concentration	Incubation	Model	Observations	References
Cyanidin	Inhibits nucleus translocation of NF-κB. Reduces COX-2 and iNOS	250 and 500 μM	24 h	SKH-1 hairless mice	Cyanidin was topically administrated, followed by exposition to UV radiation	[58]
	Reduces p-STAT3	100 and 200 μM	4 h	Peripheral blood mononuclear cells	Stimulated with IL-6	[115]
Delphinidin	Suppresses MMP9 expression, activation of NF-κB and AP-1	60 μM	24 h	MCF-7 cells	Co-treatment with Phorbol Myristate Acetate (PMA)	[59]
Epigallocatechin	Inhibits NF-κB, MMP-2, and MMP-9 activity. Reduces cytokines and chemokines production	40 μg/mL	24 h	LnCAP, DU-145, and PC-3 cells	Followed by CpG-ODN or TNFα treatment	[57]
Eriodictyol	Inhibits NF-κB pathway	100 µM	48 h	U87MG and CHG-5 cells	-	[62]
Naringenin	Down-regulates NF-κB pathway and TNF-α, IL-1β, and IL-6 production	50 mg/kg b.wt	16 weeks	Swiss albino mice	B[a]P induced lung carcinogenesis in vivo	[65]
	Down-regulates MMP2, MMP9, and p38 activity	200 and 300 µM	24 h	8901 and 8401 GBM cells	-	[86]
Apigenin	Inhibits IL-6, IL-1β, TNF-α, NF-κB, caspase-1, and NLRP3 assembly	25 µM	2 h	THP-1-derived macrophages and HEK293 cells	Followed by IL-1β or TNF-α or LPS treatment	[64]
	Reduces phosphorylation of src, JAK2, and STAT3	20–40 µM	24 h	HL60 and TF1 cells	-	[112]
	Inhibits IKK activation and suppresses NF-κB activation	20 and 50 μg/mouse/day	20 weeks	TRAMP mice, oral gavage	-	[54]
	Inhibits STAT3 activity, reduces MMP-2, MMP-9, VEGF, and TWIST expression	40 µM in cells or 150 mg/kg/day in mice	24 h in cells or 24 days in mice	A375, and G361 cells. Lung metastasis in C57BL/6 mice	-	[113]
Vitexin	Reduces phosphorylation of p38 and JNK	25, 50, and 100 μg/mL	2 h	RAW 264.7 cells	Stimulated with LPS	[87]
Luteolin	Attenuates TNF-α, IL-8, IL-6, and COX-2 expression. Decreases p-JNK 1/2. Inhibits NF-κB activation and IκB degradation	50 µM	1 h	HMC-1 cells	Stimulation with PMA plus A23187	[63]
	Deactivates STAT3 Reduces MMP2, MMP7, and MMP9 levels	80 µM	24 h	PANC-1 and SW1990 cells	-	[110]
Kaempferol	Inhibits NF-κB activity	10 µM	72 h	Jurkat cells	TNF-α treatment	[53]
	Reduces p-STAT3	75 µM	72 h	LS174 cells	Alone or combined with 5-FU	[111]
	Inhibits NF-κB activity and c-Fos expression	25, 50, or 100 µM	1 h	RAW264.7 cells	Followed by LPS treatment	[60]
Myricetin	Reduces TNF-α, IL-1β, IL-6, NF-κB, p-NF-κB, and COX-2	40 and 100 mg/kg	4 weeks	AOM/DSS-induced colitis and tumorigenesis in model mice	-	[61]
Quercetin	Down-regulates TLR4 and NF-κB pathways. Reduces secretion of IL-1β, IL-6, TNF-α, and IL-10	5 μM	4 h	A549 cells	Followed by nickel exposition	[56]
	Inhibits NF-κB nuclear translocation	50 and 100 μM	24 h	ACC-2 and ACC-M cells	-	[67]
	Down-regulates NF-κB, p-IκB-α, p-IKK-β, and cyclin D1	80 μM	24 h	HeLa cells	-	[55]
Genistein	Inhibits NLRP3 inflammasome via TGR5-cAMP	20 µM	2 h	THP-1 and U937 cells	Followed by LPS and ATP treatment	[100]
	Inhibits NLRP3 and NF-κB pathway	1, 5, and 10 μM	1 h	RAW264.7 cells	Followed by LPS treatment	[101]
	Inhibits NF-κB activity	10 μM	30–120 min	HCT116 cells	Combined LPS treatment	[52]
Taxifolin	Down-regulates NF-κB, TNF-α, COX-2, and cyclin D1	4 μg/kg	15 weeks	Swiss albino mice	Pre-treated with 1,2-Dimethylhydrazine (DMH)	[66]

**Table 4:** Molecular mechanisms of flavonoids in redox metabolism and cancer.

Redox Metabolism						
	Molecular Mechanisms	Concentration	Incubation	Model	Observations	References
Epigallocatechin	Acts as a chemosensitizer in breast cancer cells by activating NRF2 signaling	25 µM	24 h	HeLa cells	Observed in Cisplatin triple-negative cells	[167]
	Inhibits cell proliferation under hypoxia via the downregulation of HIF-1α and its downstream target gene VEGF levels	80 μg/mL	48 h	SGC7901 cells	-	[182]
Naringenin	Suppresses the early stage of colon cancer by attenuating iNOS and COX-2 levels	200 mg/kg	10 weeks	Sprague-Dawley rats injected with a carcinogen	-	[188]
Apigenin	Decreases PKM2 expression	10–60 µM	24 h	HCT116, HT29, and DLD1 cells	-	[148]
	Reduces cell viability in a dose- and time-dependent manner through CAT and GSH activity	100 μmol/L	24–48 h	HepG2 cells	Observed in doxorubicin-resistant hepatocarcinoma	[171]
	Down-regulates of HIF1α and GLUT-1 mRNA expression. Represses hypoxia-mediated induction of GLUT-1 expression. Significant reduces of the HIF1 protein level	50 µM	24 h	S2–013 and CD18 cells	-	[186]
	Translocates FOXO1 and reduces G6Pc-mRNA levels	20–100 µM	24 h	U-2 OS cells	-	[192]
Luteolin	Inhibits colon carcinogenesis through iNOS and COX-2	1.2 mg/body kg	3 weeks	Balb/c mice	-	[190]
	Regulates HIF1α -VEGF/MMP9signaling pathway through suppression of HIF1α activation	20 µM	24 h	RAW264.7 cells	-	[184]
	Induces cell apoptosis through antioxidant enzymes activity like SODs e CAT	50 µM	6 to 24 h	CH27 cells	-	[201]
	Translocates FOXO1 and reduces G6Pc-mRNA levels	10–100 µM	24 h	U-2 OS cells	-	[192]
	Increases NRF2, increases GSH content, decreases the level of GSSG, and prevents tumorigenesis	1–20 µM	8–24 h	Caco-2, HT-29, HepG2, and HEK-293 cells	-	[160]
Kaempferol	Blocks ROS generation causing cell cycle arrest at G1 and G2/M arrest involving p53 and p38	50 and 100 μM	24, 48, and 72 h	HCT116 cells	-	[195]
	Reduces the COX-2, p-AKT, and p-ERK levels, decreasing tumor growth	60 or 120 µM	48 h	MKN28 and SGC7901 cells	-	[197]
	Causes HIF1α mislocalization into the cytoplasm due to p44/42 MAPK inactivation, resulting in the suppression of HIF1 activity	5 to 50 µM	4 h	Huh7 cells	-	[185]
	Triggers ROS generation and apoptosis through reduction of the thioredoxin and SOD concentrations	50 µM	72 and 96 h	LN229, U87MG, and T98G cells	-	[194]
Myricetin	Activates NRF2 by modifying the KEAP1 protein, decreasing NRF2 ubiquitination, and increasing HO-1 levels	20 µM	24 h	HepG2 cells	-	[174]
Quercetin	Interacts directly with NRF2 increasing its half-life	0–40 µM	6 to 12 h	HepG2 cells	-	[169]
	Inhibits metastasis of cancer cells by blocking AKT/mTOR/c-Myc signaling pathway	40 µM	24 h	A431-III cells	-	[202]
	Suppresses the mobility of breast cancer by suppressing glycolysis through AKT-mTOR-PKM2	50 mg/kg twice daily	1 month	BALB/c nude mice	-	[149]
	Modulates the balance between HIF1α translation and degradation, inhibiting HIF1α protein synthesis and accumulation	100 µM	8 h	LNCaP, SkBr3, and CX-1 cells	-	[179]
Daidzein	Increases expression of QR mRNA and its activity, as well as increased NRF2/ARE binding capacity	1 and 5 µM	24 and 48 h	Hepa-1c1c7 cells	-	[191]
Genistein	Increases expression of QR mRNA and its activity, as well as increased NRF2/ARE binding capacity	1, 5, and 25 µM	24 and 48 h	Hepa-1c1c7 cells	-	[191]
	Reduces the level of methylation in the KEAP1 promoter region, leading to an increased mRNA expression, thus effectively inhibited the transcription of NRF2 to the nucleus	10 µM	48 h	A549 cells	-	[177]
	Sensitizes aerobic glycolytic cells to apoptosis by directly downregulating HIF1α, inactivating GLUT1 and HK2 to suppress aerobic glycolysis	20–80 mg/kg	21 days	Athymic BALB/C nu/nu mice	-	[187]
Vitexin	Suppresses melanoma cell growth through DNA damage by increasing ROS levels	40 or 80 mg/kg	2–3 weeks	c BALB/c female nude mice (nu/nu)	-	[178]
		5–20 µM	24 h	A375, Sk-Mel-5, and Sk-Mel-28 cells	-	
	Decreases ROS levels, increases GSH and SOD levels	10 and 100 µM	24 h	PC12 cells.	-	[200]
Taxifolin	Inhibits NF-κB-mediated Wnt/β-catenin signaling, via up-regulation of NRF2 pathway	4 μg/kg	15 to 30 weeks	Albino Swiss mice	-	[66]

**Table 5:** Molecular mechanisms of flavonoids in cell growth signaling and cancer.

Cell Growth Signaling							
	Molecular Mechanisms	Concentration	Incubation		Model	Observations	References
Cyanidin	Targets PDK1 to suppress PI3K/AKT signaling, decreasing p-AKT, p-mTOR, and PDK1 activity	40 µM	24 h	HepG2, Hep3B, HepG2/DDP, and Hep3B/DDP cells. HepG2 xenograft BALB/c mice		Reverses oxaliplatin resistance	[252]
Cyanidin-3-o-glucoside	Inhibits the growth of MDA-MB-231 cells in vivo. Inhibits EGFR/AKT signaling, and promotes EGFR degradation	150 μM and 420 mg/kg	24 h and 6 weeks	MCF-7, SK-BR-3, MDA-MB-436, BT-20, and MDA-MB-231 cells and MDA-MB-231-luc xenograft mice		-	[253]
	Increases p-ERK1/2 and p-p38 MAPK, and decreases p-AKT	5, 50 and 100 µM	48 h	Meg-01 cells		-	[254]
Delphinidin	Inhibits proliferation and decreases p-AKT, p-mTOR, p-S6K1, and p-eIF4E	20, 40, and 80 μM	48 h	MDA-MB-453 and BT474 cells		Increases p-AMPK at 80 μM	[260]
	Prevents HIF1α expression and ERK1/2, AKT, mTOR, and S6K1 phosphorylation	10, 20, and 40 μM	1h	A549 cells		Induced by cobalt chloride (200 μM) and EGF (20 ng/mL) treatments	[261]
	Decreases p-PI3K, p-AKT (Ser473), and p-ERK1/2	20, 40, and 60 μM in cell culture. 1 or 2mg in xenograft BALB/c mice	48 h	NCI-H441 and SK-MES-1 cells and NCI-H441 xenograft mice		Decreased tumor volume in the NCI-H441 xenograft model	[212]
	Decreased p-AKT, p-S6K1, and p-ERK	0.1, 1 and 10 μM	24 h	ES2 cells		Delphinidin exhibits the same effects as cisplatin and paclitaxel	[262]
	Decreased cell proliferation in a dose-dependent manner and decreased p-ERK1/2, p-S6K1, p-S6, and p-AKT	0.1, 1 and 10 μM	30 min	SKOV3 cells		Sensitizes to paclitaxel treatment	[263]
Epigallocatechin gallate (EGCG)	Decreases p-mTOR and p-AKT and increased PTEN	40 µg/ml	48 h	PANC-1 and BxPC-3 cells		-	[215]
	Decreases p-IGF1R, p-ERK1/2, and p-AKT in SW837 cells.	25 μg/mL in cell culture and tap water containing 0.1% or 1% EGCG in xenograft mice model.	3,6,12 and 24 h in cell culture and 35 days in xenograft mice model.	SW837 cell line and xenograft SW837 BALB/c nude mice.		Decreases tumor volume while decreases p-AKT and p-ERK1/2 at 0.1 and 1% of EGCG in xenograft BALB/c mice	[250]
	Inhibits the proliferation in a dose-dependent manner. Decreases p-AKT and p-mTOR	10, 20, 40, and 80 μM	24, 48, and 72 h	PANC-1 cells		-	[272]
	Prevents AKT phosphorylation (Ser473) induced by IGF-1 treatment in A549, MDA-MB-231, and LnCAP cells, but not in PC-3	25 μM	1 h	MDA-MB-231, PC-3, LnCAP, and A549 cells		Decreases MDA-MB-231 and A549 cell proliferation in a dose-dependent manner after 72 h	[251]
	Decreases HuH7 cell proliferation in a dose-dependent manner. Decreases VEGF secretion. Decreases tumor volume and p-VEGF, p-ERK1/2 and p-AKT	0–100 µg/mL in cell culture/tap water containing 0.01 or 0.1% EGCG in mice xenograft model	24 or 48 h/5 weeks	HuH7 cells and mice BALB/c xenograft model		-	[273]
Eriodictyol	Decreases p-PI3K, p-mTOR, and p-AKT	25, 50 and 100 μM	48 h	A549 and FR2 cells		-	[209]
	Decreases p-PI3K and p-AKT	25, 50 and 100 μM	48 h	U87-MG and CHG-5 cells		-	[62]
Naringenin	Decreases p-AKT (Ser473), p-MEK1/2, and p-MAPK after insulin stimuli. Inhibits glucose uptake	100 μM	15 min	T47D and MCF-7 cells		-	[248]
	Decreases p-AKT	200 μM	24 h	THP-1 cells		-	[274]
	Decreases p-AKT	20, 40, or 80 μM	24, 48 and 72 h	SGC7901 cells		-	[275]
	Decreases p-AKT	40 µM	48 h	SGC7901 cells		Combined with 5 µM ABT-737	[249]
	In JAR cells, increases p-ERK1/2, p-AKT, and p-S6K1, but decreases p-S6. In JEG cells, increases p-ERK1/2, p-S6K1, but decreases p-S6 and p-AKT	12.5–100 μM	48 h	JAR and JEG-3 cells		-	[276]
Apigenin	Prevents AKT phophorylation	40 μM	2 h	MDA-MB-231 cells		Induced by Hepatocyte growth factor (40 ng/mL)	[243]
	Prevents p-AKT (Ser473) and p-GSK3b (Ser9) during hypoxia. Inhibits HIF1α activity and decreases VEGF mRNA levels during hypoxia	25, 50 and 100 μM	1 h	PC3–M cells		-	[277]
	Increases p-AKT (Ser473), p-ERK1, and p-ERK2 in dorsolateral prostate of TRAMP mice decreases IGF-I and increases IGFBP-3 in the serum and the dorsolateral prostate	20 μg and 50 μg/day	20 weeks	C57BL/TGN TRAMP mice		Apigenin also inhibits tumor growth and metastasis	[278]
	Increases p-AMPK (Thr172) and decreases p-AKT (Ser473), LKB1, p-S6K1 (Thr389), p-4EBP1 and 4EBP1	20 μM	24 h	HaCaT cells		-	[225]
	Decreases p-AKT, p-mTOR and p-ERK1/2	100 μM	24 h	A375 and C8161cells		-	[244]
Luteolin	Decreases p-AKT (Ser473), p-S6K1, and p-S6.	10, 25, and 50 μM	48 h	T24 and 5637 cells		Decreases tumor growth in the xenograft model	[245]
	Decreases p-AKT, p-mTOR, p-S6K1 and increases p-ERK1/2. Decreases expression of SREBP1, SREBP2, and SREBP cleavage-activating protein (SCAP) mRNAs and protein expression	5, 10 and 20 μM	48 h	JAR and JEG-3 cells		Exhibits synergistic effects with etoposide, cisplatin, and paclitaxel	[228]
	Decreases p-AKT, p-S6, and p-4EBP1 in NCI–H1975 cells	10, 30, 50, 80 and 100 μM	24 h	A549, HCC827, and NCl-H1975 cells		-	[226]
	Decreases p-IGFR, p-AKT, and p-mTOR	5, 10 and 20 μM	24 h	U251MG and U87MG cells		-	[279]
Vitexin	Decreases p-AKT, p-mTOR and p-PI3K	20 μM	48 h	A549 cells		-	[207]
	Increases p-AMPK and decreases p-PI3K, p-AKT, and p-mTOR	20 and 40 μM	24 h	ACHN and OS-RC-2cells		-	[206]
Myricetin	Decreases p-AKT (Ser473) and p-S6K1	5–20 μM	24 h	A2780/CP70 and OVCAR-3 cells		-	[241]
	Increases p-AKT, p-S6K1, p-S6, and p-ERK1/2	20, 50 and 100 μM		D-17 canine osteosarcoma cells		-	[270]
	Increases p-AKT, p-ERK1/2 and p-p90RSK	5, 10 and 20 μM		JAR and JEG-3 cells		Exhibits synergistic antiproliferative effects with cisplatin and etoposide	[271]
	Decreases AKT, p-AKT (Ser473), and EGFR expression. 200 uM decreases K-ras and Raf-1, ERK and p-ERK	25–200 μM	24 h	DBTRG cells		-	[242]
	Decreases p-ERK and ERK, AP-1 and MEK1 activity	5–20 μM	1 h	JB6 P+ and H-Ras-transformed JB6 P+ cells		Treatment combined with 12-O-tetradecanoylphorbol-13-acetate (TPA) 20 ng/mL)	[135]
Kaempferol	Increases PTEN while decreases p-PI3K, p-AKT, p-mTOR and p-S6K1	50 μM	24 h	HepG2 cells		-	[213]
	Increases p53 and PTEN while decreases PI3K and AKT mRNA expression	25, 50 and 100 μM	24, 48, and 72 h	HeLa cells		-	[214]
	Increases PTEN and decreases p-AKT	40 μM	48h	EJ cells		-	[236]
	Decreases p-AKT (Ser473 and Thr308).	50 μM	48 and 72 h	K562 and U937 cells		-	[235]
	Decreases p-AKT (Ser473)	25–100 μM	24 h	RCC 786-O cells		-	[237]
	Inhibits p-AKT (Ser473) and increases p-MEK2 and p-MAPK	35–70 μM	48 h	A549 cells		-	[238]
Quercetin	Decreases the expression of SREBPs and nuclear chSREBP	25 mM	4 h	C6 cells		-	[229]
	Decreases p-AKT and p-mTOR	25 and 50 μM	24 h	SMMG-7721 and BEL-7402 cells		-	[232]
	Suppresses cell invasion and migration of breast cancer while decreases p-mTOR, p-AKT, and p-S6K1. Decreases tumor growth in xenograft model though decreases of VEGF+ cells, p-AKT, and PKM2	30 µM in cell culture. 50 mg/kg in xenograft model	24 h/4 weeks	MCF7 and MDA-MB-231 cells and MCF7 xenograft BALB/C mice		-	[149]
	Decreases tumor growth	15 and 45mg/kg	13 weeks	GFP-MDA-MB-231 xenograft SCID mice		-	[224]
	Decreases p-4EBP1, p-S6K1, p-AKT	15 μM	15 min	MDA-MB-231and MDA-MB-435 cells		-	[224]
	Increases p-AMPK, Sestrin2, p53 and decreases p-mTOR	25 and 50 μM	6 h	HCT116 cells		-	[280]
Quercetin-6-C-β-d-glucopyranoside	Suppresses proliferation through decreases of p-AKT and p-mTOR	60 μM (PC-3) and 100 μM (DU-145)	24 and 48 h	PC-3 and DU-145 cells		-	[233]
Daidzein	Decreases p-PI3K, p-AKT, and p-mTOR	10 µM CC and 50 µM Dadizein	48 h	MCF-7 and MDA MB-231 cells		Treatments separately or together	[210]
	Decreases p-PI3K, p-AKT, and p-GSK3β	10 and 50 µM	24 h	SKOV-3, A2780CP, and OVCAR-3 cells		-	[211]
	Anti-proliferative activity against MCF-7 cells and in MDA-MB-231. Inhibits proliferation and invasion	MCF-7 cells at 66.98 ± 4.87 μM and MDA-MB-231 at 93.75 ± 5.15 μM		MCF-7 and MDA-MB-231 cells		-	[240]
Genistein	Decreases p-AKT and p-mTOR	40 µM	12 h	human colon cancer HT-29		Treatment combined with indol-3-carbinol	[255]
	Enhances the antitumor activity of cisplatin and reduces p-mTOR, p-p70S6K1, p-4E-BP1, and p-AKT	25 μM	24 h	HeLa cells.		Sensitizes cells to cisplatin treatment.	[256]
	Decreases cell viability, p-PI3K, and p-AKT	160 μM	12 and 48 h	Human Bladder Cancer T24 Cells		-	[257]
	Decreases p-EGFR and p-AKT (Ser473) and increases p-ERK1/2	50-200 µM	24 h	The HuCCA-1 and RMCCA-1 human intrahepatic CCA cell lines		-	[258]
	Decreases cell viability, p-AKT, and p-HIF1α	50 µM	48 h	A549 human lung carcinoma cells		-	[259]
S-Equol (a metabolite of Daidzein)	Decreases p-AKT, p-ERK, and p-FOXO3a in vitro. Inhibits the growth of PC-3 xenograft tumors in BALB/c nude mice	100, 150, and 200 µM	24 h	LnCaP, DU-145, and PC-3 cells		-	[269]
Taxifolin	Decreases p-PI3K, cell viability and tumor growth	25, 50 and 100 μM in cells/1mg/kg in mice	24 h/25 days	A549 and H1975 cells and A549 Xenograft BALB/c nude nude		-	[208]
	Taxifolin binds to EGFR and PI3K and decreases its activity. Decreases p-AKT (Thr308 and Ser473), p-S6K1, and p-ERK1/2	20, 40, and 80 μM	24 h	JB6 P+ cells		-	[216]
	Decreases p-AKT (Ser473) in cell lines and tumor growth in a xenograft model	25 and 50 μM	48 h	U2OS and Saos-2 cells and U2OS xenograft BALB/c nude mice		-	[246]

**Table 6:** Molecular mechanisms of flavonoids in autophagy and cancer.

Autophagy						
	Molecular Mechanisms	Concentration	Incubation	Model	Observations	References
Delphinidin	Increases LC3-II, Atg5/12, and p-ULK	80 µM	24 h	HER-2 (breast)	-	[260]
	Stimulates the formation of autophagosomes (decreases p62 and increases LC3-II)	100 µM	24 h	U20S cells	-	[320]
	Increases LC3-II	20 µM	48 h	A549 cells	-	[321]
Epigallocatechin	Increases Beclin1, LC3-II, p62, and Atg5/12/16	250 µg/mL	24 h	A375 cells	-	[322]
	Increases LC3-II	12.5 µM	24 h	HTC-116 cells	-	[323]
	Increases LC3-II	150 µM	24 h	A549 cells	Sensibilization to γ-ray irradiation	[324]
	Increases LC3-II	100 µM	48 h	HT-29 and DLD-1 cells	-	[325]
	Increases LC3-II, Beclin1, and Atg5/7/12	100 µM	24 h	CAR cells	-	[326]
Naringin	Increases Beclin1 and LC3-II	2 mM	24 h	AGS cells	-	[319]
Apigenin	Increases Beclin1 and LC3-II	25, 50, 100, and 200 µM	36 h	U251, U118, and U87 cells	-	[312]
	Increases Beclin1 and LC3-II	80 µM	12 h	HepG2 cells	-	[313]
	Stimulates the formation of autophagosomes (decreases p62 and increases LC3-II)	90 µM	24 h	HepG2 cells	-	[314]
	Stimulates the formation of autophagosomes (decreases in p62 and increase in LC3-II), increase in Beclin1, and Atg5	10, 20, and 40 µM	24 h	HepG2	-	[315]
Luteolin	Increases Beclin1 and LC3-II	80 µM	24 h	HepG2 cells	-	[316]
	Stimulates the formation of autophagosomes (decreases p62 and increases LC3-II)	100 µM	48 h	Hep 3B cells	-	[317]
	Stimulates the formation of autophagosomes (decreases p62 and increases LC3-II)	20 µM	24 h	MET4 cells	-	[318]
	Stimulates the formation of autophagosomes (decreases p62 and increases LC3-II)	100 µM	24 h	MET4 cells	-	[311]
Kaempferol	Increases Beclin1, LC3-II, and in Atg5, 7, 12	75 µM	24 h	HepG2 cells	-	[305]
	Inhibits autophagosome formation (accumulation of LC3-I and p62). Reduces Beclin1	20 µM	48 h	SKVCR cells	-	[306]
	Increases Beclin1. Stimulates the formation of autophagosomes (decreases p62 and increases LC3-II). Increases Atg5	25 µM	24 h	HT-29 cells	-	[307]
	Stimulates the formation of autophagosomes (decreases p62 and increases LC3-II). Increases Atg5, 7	100 µM	24 h	HepG2 e Hh7 cells	-	[308]
Myricetin	Increases Beclin1. Stimulates the formation of autophagosomes (decreases p62 and increases LC3-II)	60 µM	24 h	MCF-7/MDA-MB-231cells	-	[309]
	Increases Beclin1 and LC3-II	100 µM	24 h	HCT116 and SW620 cells	-	[310]
	Increases Beclin1. Stimulates the formation of autophagosomes (decreases p62 and increases LC3-II)	100 µM	24 h	SK-MEL-28 cells	-	[311]
Quercetin	Increases Beclin1. Stimulates the formation of autophagosomes (decreases p62 and increases LC3-II)	40 µM	24 h	HepG2 cells	-	[303]
	Increases Beclin1 and LC3-II	100 µM	24 h	PC-3 cells	-	[304]
	Increases Beclin1 and LC3-II	30 µM	24 h	MCF-7 and MDA-MB 231 cells	-	[149]
Genistein	Increases LC3	100 µM	72 h	MCF-7 cells	-	[327]
	Increases Beclin1	500 ppm	55 days	Xenografts of breast tumors	-	[328]
	Increases Beclin 1. Stimulates the formation of autophagosomes (decreases p62 and increases LC3-II)	60 µM	24 h	A549 cells	-	[329]
	Increases LC3-II and Beclin1	100 µM	24 h	MIA-PaCa 2 cells	-	[330]
	Increases Beclin 1 and p62	40 µM	24 h	A549 cells	-	[331]
Quercetin	Increases Atg5. Stimulates the formation of autophagosomes (decreases p62 and increases LC3-II)	10, 20, and 40 µM	24 h	MDA-MB-231 cells	-	[302]
Apigenin (Apigenin-7-methyl ether)	Stimulates the formation of autophagosomes (decreases in p62 and increase in LC3-II), increases in Beclin1, and Atg5	20, 40, and 80 µM	24 h	MDA-MB-231 cells	-	

**Table 7:** Molecular mechanisms of flavonoids in apoptosis, cell cycle, DNA repair, and senescence.

Apoptosis						
	Molecular Mechanisms	Concentration	Incubation	Model	Observations	References
Cyanidin	Up-regulates cleaved Caspase 3	50–100 µg/mL	24 h	Jurkat cells	-	[368]
	Up-regulates p53 and caspase 3	50–300 µg/mL	24 h	HeLa cells	-	[342]
	Up-regulates p53 and caspase 3	100–400 µg/mL	24 h	HeLa cells	-	
**Epigallocatechin**	Down-regulates Mcl-1, c-FLIP, and Bcl-2	50 µg/mL	48 h	Renal Cell Carcinoma (786-O)	The treatment was combined with 200 ng/mL of TRAIL	[351]
**Eriodictyol**	Down-regulates Bcl-XL	25–100 µM	48 h	U87MG and CHG-5 cells	-	[62]
	Up-regulates BAX/Bcl-2 ratio	50–100 µM	24/48 h	A549 cells	-	[209]
**Naringenin**	Down-regulates Bcl-2	150–200 µM	24 h	HepG2 cells	-	[345]
	Up-regulates p53, Caspase 3 and BAX	150–200 µM	24 h	HepG2 cells	-	
**Apigenin**	Down-regulates Mcl-1 and c-FLIP	50 and 100 µM	8 h	Human leukemic T cell line Jurkat HTLV-1-associated ATL cells	-	[354]
	Up-regulates p53	50 and 100 µM	16 h	ATL cells	-	
	Down-regulates Bcl-2	20 µM	24 h	PC-3 cells	-	[347]
	Up-regulates BAX	20 µM	24 h	PC-3 cells	-	
	Up-regulates PARP cleaved	50 µg/day	8 weeks	Athymic nude mice	-	
**Luteolin**	Up-regulates BAX and Caspase 3	10–30 µM	48 h	MDA-MB-231 cells	-	[344]
	Down-regulates Bcl-2	10–30 µM	48 h	MDA-MB-231 cells	-	
**Kaempferol**	Increases cleaved caspase 3	10 µM	72 h	Miapaca-2 cells	Tre treatment was compared with Gefetinib action	[341]
	Increases cleaved caspase 3	100 µM	72 h	Panc-1 cells	Tre treatment was compared with Gefetinib action	
**Myricetin**	Down-regulates Bcl-2	50 and 100 µM	24 h	HCT-15 cells	-	[346]
	Up-regulates BAX	50 and 100 µM	24 h	HCT-15 cells	-	
**Vitexin**	Up-regulates cleaved Caspase-3 and Caspase-9	10–40 µM	24 h	ACHN and OS-RC-2 cells	The treatment was compared with Metformin and Rapamycin action	[206]
	Down-regulates Bcl-2 and up-regulates BAX and cleaved Caspase-3 and Caspase-9	10–40 µM	48 h	A549 cells	-	[207]
**Quercetin**	Down-regulates c-FLIP	200 µM	12 h	HepG2 cells	-	[369]
	Down-regulates c-FLIP	50, 100 and 200 µM	12 h	SNU-475 cells	-	
	Up-regulates cleaved PARP and p53	20 µM	24 h	Leukemia cell (Nalm6)	-	[353]
	Down-regulates Mcl-1	20 µM	24 h	Leukemia cell (Nalm6)	-	
	Up-regulates BAX, Caspase 3 and p53	80 µM	24 h	HeLa cells	-	[55]
**Daidzein**	Down-regulates Bcl-2	90 µM	24 h	BEL-7402 cells	-	[352]
	Up-regulates cleaved PARP	25–100 µM	48 h	Choriocarcinoma cell line (JAR)	-	[349]
	Down-regulates Bcl-2/BAX ratio	25–100 µM	48 h	Choriocarcinoma cell line (JEG-3)	-	
**Genistein**	Up-regulates Caspase 3	30–70 µM	48 h	HT29 cells	-	[343]
	Up-regulates BAX	20–50 µM	48 h	HL-60 cells	-	[348]
	Down-regulates Bcl-2	40–50 µM	48 h	HL-60 cells	-	
	Up-regulates BAX and BAK	0.2–0.4 mg/kg	23 days	Athymic nude mice	-	
**Taxifolin**	Up-regulates BAK	40–60 µM	24 h	HCT116 cells	-	[350]
	Down-regulates Bcl-2, Bcl-xL, and Bid	40–60 µM	24 h	HCT116 cells	-	
**Cell Cycle**						
	**Molecular mechanisms**	**Concentration**	**Incubation**	**Model**	**Observations**	**References**
**Anthocyanidins**	Up-regulates p21	10–100 µg/mL	24 h	Jurkat cells	-	[368]
	Down-Downregulates Cyclin D1	400 µg/mL	24 h	HeLa cells	-	[342]
	Down-regulates Cyclin D1	50–300 µg/mL	24 h	HeLa cells	-	
**Luteolin**	Down-regulates Cyclin D1	10–30 µM	24 h	MDA-MB-231 cells	-	[344]
	Up-regulates p21	10–30 µM	24 h	MDA-MB-231 cells	-	
**Kaempferol**	Down-regulates Cyclin D1, Cyclin A, and Cyclin E	60 µmol/L	6 h	HT-29 cells	-	[196]
**Quercetin**	Up-regulates p21	80 µM	24 h	HeLa cells	-	[55]
**Daidzein**	Down-regulates cyclin D, CDK2, CDK1, and CDK4	100 µM	72 h	MDA-MB-453 and MCF-7 cells	-	[370]
Genistein	Up-regulates p53 and p21	50–100 µM	72 h	HCT116 cells	-	[358]
**Taxifolin**	Up-regulates p21	40–60 µM	24 h	HCT116 cells	-	[350]
	Down-regulates Cdk-2, Cdk-4, Cdk-6, Cyclin D, Cyclin A, Cyclin B	40–60 µM	24 h	HCT116 cells	-	
**DNA Repair**						
	**Molecular mechanisms**	**Concentration**	**Incubation**	**Model**	**Observations**	**References**
**Catechin**	Down-regulates CHK1	5–10 µM	30 min	MDA-MB-231 cells	-	[360]
**Epigallocatechin**	Down-regulates CHK1/2	10–50 µM				
**Naringenin**	Down-regulates CHK1/2	50 µM				
**Kaempferol**	Down-regulates CHK1/2	50 µM				
**Genistein**	Down-regulates CHK1/2	5 µM				
**Senescence**						
	**Molecular mechanisms**	**Concentration**	**Incubation**	**Model**	**Observations**	**References**
**Quercetin**	Increases p16	25 μg/mL	48 h	Colo-320 and Colo-741 cells	-	[365]
**Epigallocatechin**	Telomerase shortening and increase of β-galactosidase	10 μg/mL	98 days	U251 cells	-	[366]
**Apigenin**	Increasesβ-galactosidase	Above 25 μM	6 days	HCT-15 cells	-	[367]
